# The C-terminal tail extension of myosin 16 acts as a molten globule, including intrinsically disordered regions, and interacts with the N-terminal ankyrin

**DOI:** 10.1016/j.jbc.2021.100716

**Published:** 2021-04-28

**Authors:** Elek Telek, Kristóf Karádi, József Kardos, András Kengyel, Zsuzsanna Fekete, Henriett Halász, Miklós Nyitrai, Beáta Bugyi, András Lukács

**Affiliations:** 1 Department of Biophysics, Medical School, University of Pécs, Pécs, Hungary; 2 MTA-PTE Nuclear-Mitochondrial Interactions Research Group, Pécs, Hungary; 3 Szentágothai Research Center, Pécs, Hungary; 4 Department of Biochemistry, Institute of Biology, Eötvös Loránd University, Budapest, Hungary

**Keywords:** myosin 16, C terminus of myosin 16, intrinsically disordered, molten globule, tryptophan fluorescence, backfolding interaction, ANS, 1-anilino-naphthalene-8 sulfonic acid, BeStSel, Beta Structure Selection, BME, β-mercaptoethanol, G-actin, globular actin, GuHCl, guanidine hydrochloride, IDP, intrinsically disordered protein, IDR, intrinsically disordered region, I-TASSER, Iterative Threading Assembly Refinement, Myo16, myosin 16, Myo16Ank, myosin 16 ankyrin domain, Myo16IQ, myosin 16 IQ motif, Myo16Tail (−IQ), Myo16Tail without the IQ motif, Myo16Tail, myosin 16 C-terminal tail, NHM, neuronal tyrosine-phosphorylated adaptor for PI3K homology motif, PI3K, phosphoinositide 3-kinase, Pro-rich, proline-rich, PTMs, post-translational modifications, TCSPC, time-correlated single-photon counting, WRC, WAVE1 regulatory complex

## Abstract

The lesser-known unconventional myosin 16 protein is essential in proper neuronal functioning and has been implicated in cell cycle regulation. Its longer Myo16b isoform contains a C-terminal tail extension (Myo16Tail), which has been shown to play a role in the neuronal phosphoinositide 3-kinase signaling pathway. Myo16Tail mediates the actin cytoskeleton remodeling, downregulates the actin dynamics at the postsynaptic site of dendritic spines, and is involved in the organization of the presynaptic axon terminals. However, the functional and structural features of this C-terminal tail extension are not well known. Here, we report the purification and biophysical characterization of the Myo16Tail by bioinformatics, fluorescence spectroscopy, and CD. Our results revealed that the Myo16Tail is functionally active and interacts with the N-terminal ankyrin domain of myosin 16, suggesting an intramolecular binding between the C and N termini of Myo16 as an autoregulatory mechanism involving backfolding of the motor domain. In addition, the Myo16Tail possesses high structural flexibility and a solvent-exposed hydrophobic core, indicating the largely unstructured, intrinsically disordered nature of this protein region. Some secondary structure elements were also observed, indicating that the Myo16Tail likely adopts a molten globule–like structure. These structural features imply that the Myo16Tail may function as a flexible display site particularly relevant in post-translational modifications, regulatory functions such as backfolding, and phosphoinositide 3-kinase signaling.

Myosins form a large and versatile superfamily of actin-based motor proteins that convert the chemical energy of ATP hydrolysis into mechanical force required for motion along actin filaments (1). They are expressed in all eukaryotic organisms (2) and play essential roles in a variety of cellular processes, including organelle trafficking, cytokinesis, cell shape maintenance, or muscle contraction (3, 4, 5, 6, 7). A novel unconventional myosin was described in 2001 by Patel *et al*. (8), which was originally named as myr8 (eighth unconventional myosin from rats) but later was designated as a new class: myosin XVI. Two myosin 16 (Myo16) splice variants have evolved: Myo16a, the shorter, cytoplasmic isoform and Myo16b, the predominant, longer isoform having an additional 590 amino acid extension on its C terminus. The unconventional Myo16 has an N-terminal premotor extension called the ankyrin domain composed of eight ankyrin repeats (Myo16Ank), followed by a conserved motor domain, an IQ motif in the neck region and a unique C-terminal tail extension in the Myo16b isoform (Myo16Tail) (Fig. 1).

**Figure 1:**
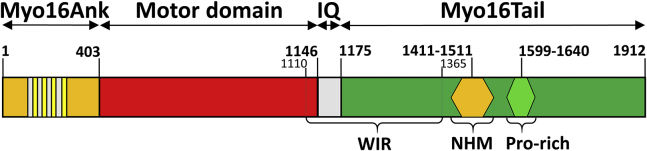
**Domain organization of Myo16b.** The numbers indicate the amino acid positions corresponding to the rat Myo16 (*Rattus norvegicus*, accession number: Q9ERC1). Myo16b (1912 aa, 210.56 kDa) is composed of an ankyrin repeat domain (Myo16Ank), a conserved motor domain followed by a single IQ motif and a C-terminal tail extension (Myo16Tail) containing a WAVE1 interacting region (WIR), a neuronal tyrosine-phosphorylated adaptor for phosphoinositide 3-kinase (NYAP) homology motif (NHM) and a proline-rich region. Myo16, myosin 16.

The tail extensions are highly diverse among myosin classes both from structural and functional aspects. Some myosin tails contain α-helical regions and can form coiled-coil structure, allowing the myosin to dimerize (9), whereas other myosins are monomers containing functional domains, for example, Src homology 3, GTPase-activating protein, four-point-one, ezrin, radixin, moesin, or pleckstrin homology domains on their tail (10). This structural diversity can lead to numerous intracellular activities and functions, such as cargo binding, dimerization, cellular localization, anchoring-tethering, protein–protein interaction, kinase activity, or autoregulation (11, 12, 13, 14).

In mammals, Myo16 is expressed predominantly in the embryonic and adult brain peaking during the 1 to 2 postnatal weeks in rats, and in a lesser amount, it can be found in some peripheral tissues. The expression of Myo16 coincides with neuronal cell migration, axonal extension, and dendritic elaboration (8). The N-terminal Myo16Ank interacts with the protein phosphatase 1 catalytic subunit and regulates its phosphatase activity (15). The protein phosphatase 1 catalytic subunit is involved in the control of synaptic plasticity, the mechanism of learning and memory (16). Myo16 is a component of the neuronal phosphoinositide 3-kinase (PI3K) signaling pathway, in which it is phosphorylated by the Src family of tyrosine kinase Fyn at its C-terminal tail (17). Phosphorylated Myo16Tail interacts with PI3K and the WAVE1 regulatory complex (WRC) simultaneously. Thereby, Myo16 can mediate actin cytoskeleton remodeling through the WRC–actin-related protein 2/3 complex (17). In line with this, Myo16 downregulates actin dynamics at the postsynaptic side of dendritic spines of Purkinje cells. In addition, Myo16 is also implicated in the organization of presynaptic axon terminals of parallel fibers (18, 19). Comprehensively, Myo16 seems to be important in the regulation of the morphological and functional features of parallel fiber-Purkinje cell synapses. Nuclear localization of Myo16b was observed in the mouse cerebellum (P23, 31) *in vivo* (8, 20), which is attributed to the C-terminal tail, although this extension does not contain any typical nuclear localization sequence. Overexpression of Myo16 in COS7 cells delays the progression through the S phase, suggesting its role in cell cycle regulation (20). The motor domain function of Myo16 has not been described yet. The above findings suggest that Myo16 has a crucial role in different aspects of neuronal functioning. In addition, genetic alterations of *MYO16* were found to be involved in neurodegenerative disorders, including schizophrenia, autism spectrum disorder, bipolar disorder subtype II, and major depressive disorder (21, 22, 23), which underlines its important role in the proper functioning of the nervous system.

Myo16b has a multifaceted C-terminal extension consisting of a WAVE1 interacting region, a neuronal tyrosine-phosphorylated adaptor for PI3K homology motif (NHM) (17), a proline-rich (Pro-rich) region, and a distal C-terminal sequence element, which is presumed to be responsible for the nuclear localization of the protein through an atypical way (20) (Fig. 1). Besides the diversity in functional motifs, the C terminus of Myo16 is supposed to have a disordered structure (24). Disordered proteins and disordered regions lack a unique 3D structure in their native, functional state (25, 26, 27); therefore, these proteins or regions were named as intrinsically disordered proteins (IDPs) or intrinsically disordered regions (IDRs), respectively (27). IDPs can be classified by the level of the structural disorder, such as molten globules, pre-molten globules, and random coils (25). The main attributions of IDPs and IDRs are the irregular amino acid composition (high content of disorder-promoting and low content of order-promoting residues) resulting in a high net charge accompanied by electrostatic repulsion and low hydrophobicity, which precludes the formation of globular structure (28). The propensity of post-translational modifications (PTMs) is an important property of IDPs, which requires site accessibility; therefore, PTM sites are located particularly in disordered regions, providing a relatively large surface on the protein. IDPs have been suggested to be enriched in phosphorylation sites (29). These structural properties have functional advantages in IDPs and IDRs, attributing structural flexibility and thus plasticity to adapt to contextual changes provided by low affinity and high specificity of binding as well as interaction with extended partners or environmental factors (25, 30, 31, 32, 33). IDPs and IDRs are involved in an extended range of biological processes, such as cell cycle regulation, recognition, signal transduction, scaffolding, transcription, or chaperoning (26, 34, 35, 36, 37). Some of these functions have been already described in connection to Myo16b (17, 20).

Here, we focused on the Myo16Tail to characterize its conformational dynamics and structural properties with the combination of sequence-based prediction, fluorescence, and CD spectroscopic approaches. For the first time, we described the expression and purification of Myo16Tail. *In silico* bioinformatic analysis was performed to investigate the structural behavior of Myo16Tail at the level of the primary amino acid sequence. The *in silico* findings were further addressed and confirmed experimentally.

## Results

### Sequence-based in silico analysis of Myo16Tail predicts disordered regions

Myo16Tail (1146–1912 amino acids, *Rattus norvegicus*, UniProt: Q9ERC1) consists of the 30 amino acids of an IQ motif, an NHM which is important in the neuronal PI3K signaling pathway, a Pro-rich region, and a very C-terminal sequence element (Fig. 1). To begin the characterization of the conformational properties of Myo16Tail, its primary sequence was analyzed. The analysis revealed that the sequence is dominated by disorder-promoting amino acids (A, G, R, D, H, Q, K, S, E, P) contributing about 68% to the total number of residues, whereas the order-promoting amino acids (W, F, Y, I, M, L, V, N, C, T) are present at only around 32%. It is noteworthy that the proportion of the well-known α-helical structure-breaker proline (38) is 15% of the total amino acid content; moreover, in the Pro-rich motif, it is as high as 30.7%.

**Figure 2:**
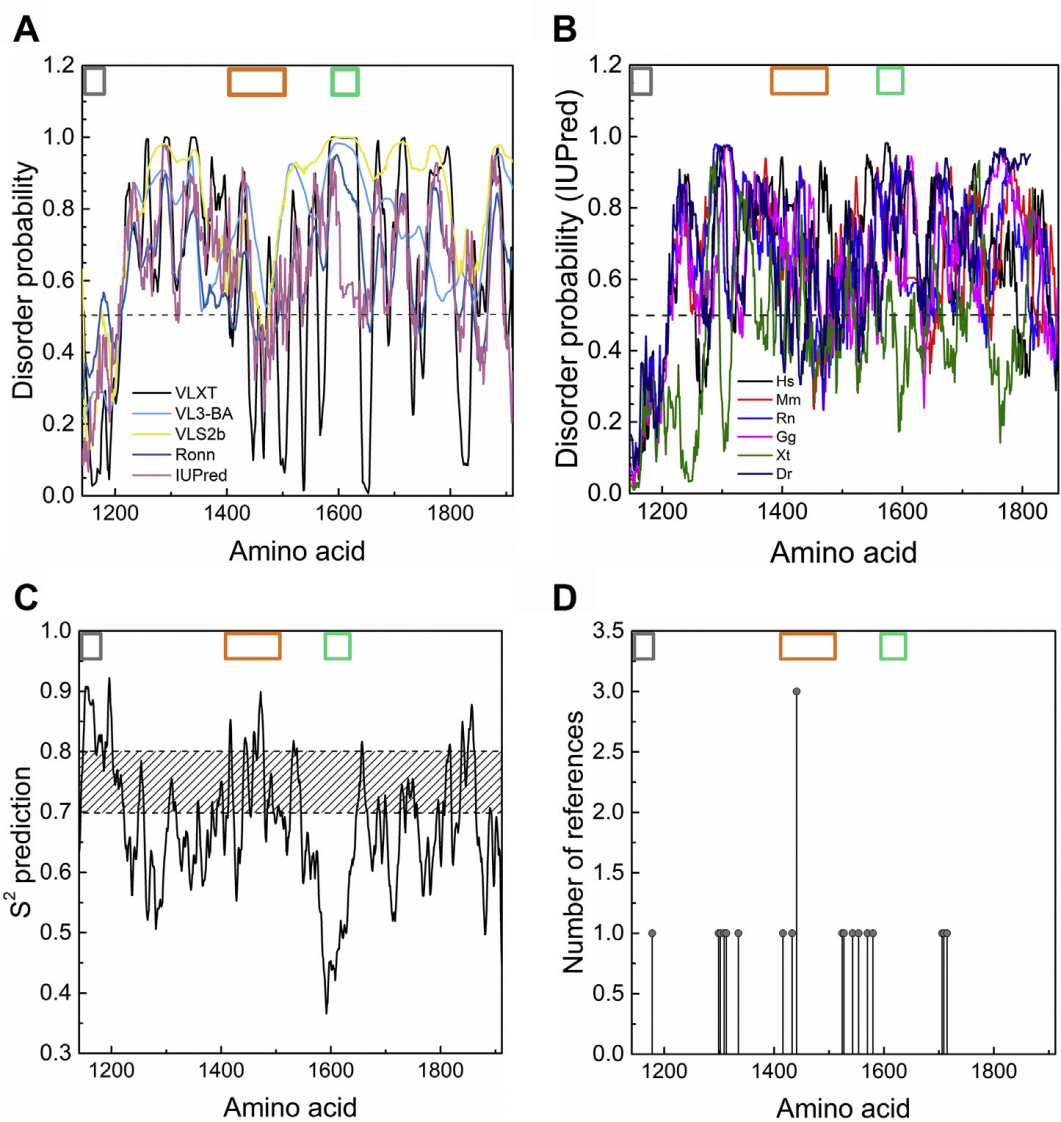
**Secondary structure prediction and dynamic propensity analysis of Myo16Tail.** The known regions of Myo16Tail are shown in *gray* (IQ), *orange* (NHM), and *green* (proline-rich) *boxes*. *A*, disorder prediction of Myo16Tail using the combination of VLXT (*black*), VL3-BA (*light blue*), VSL2b (*yellow*), RONN (*dark blue*), and IUPred (*pink*) servers. The *horizontal dashed black line* indicates the threshold at 0.5 value of disorder probability, above which the residue has more than 50% probability of being disordered. *B*, disorder conservation prediction of vertebrate class representatives of Myo16Tail by using IUPred. The following species sequences were used: *Homo sapiens (Hs*): Q9Y6X6, *Mus musculus (Mm)*: Q5DU14, *Rattus norvegicus (Rn)*: Q9ERC1, *Gallus gallus (Gg)*: XP_416950.3, *Xenopus tropicalis (Xt*): A0A5G3IJG7, and *Danio rerio (Dr)*: F1QE80. *C*, analysis of the dynamic characteristics of Myo16Tail using DynaMine. Low S^2^_pred_ values (<0.7) indicate backbone flexibility, and high S^2^_pred_ values (>0.8) suggest a rigid protein structure. The transient range (diagonally striped) indicates that in this zone, proteins have context-dependent dynamics. *D*, post-translational modification site prediction of Myo16Tail using PhosphoSitePlus. The prediction is based on the number of references. Myo16Tail, myosin 16 C-terminal tail; NHM, neuronal tyrosine-phosphorylated adaptor for PI3K homology motif.

Disorder prediction of rat Myo16Tail was performed with the combination of disorder predictors, including VLXT (39, 40), VL3-BA (41), VSL2b (42), Ronn (43), and IUPred (44, 45, 46) (Fig. 2*A*). Only the IQ motif was found to be relatively well ordered as expected (47); in contrast, the C-terminally located regions are characterized by a relatively high disorder probability. This analysis indicates that Myo16Tail is enriched in IDP regions. For additional confirmation, we analyzed the disorder probability of sequences of different vertebrate representatives of Myo16Tail using IUPred: *Homo sapiens*, *Mus musculus*, *R. norvegicus*, *Gallus gallus*, *Xenopus tropicalis*, *Danio rerio*. Based on the IUPred prediction, the sequences of representatives display similarly high disorder probability, suggesting that the intrinsically disordered structure is conserved in Myo16Tail (Fig. 2*B*). In line with this, we analyzed the conservation of the Myo16Tail sequence from different vertebrate classes in multiple sequence alignment by using Clustal X (48) (Fig. S1). The analyses showed substantial amount of conservation of Myo16Tail in the IQ and NHM motifs but not in the Pro-rich region. In addition, other regions also display a considerable number of conservations, which suppose that the predicted disordered segments are mostly conserved, suggesting that the intrinsically disordered nature of Myo16Tail is conserved through the evolution (Fig. S1). Because Myo16Tail might be involved in neurodegenerative disorders (21, 22, 23), the conserved nature of the Myo16Tail structure can have significance in the functioning of the human Myo16.

The Myo16Tail sequence was further assessed with DynaMine (49, 50) to characterize the protein backbone flexibility (Fig. 2*C*). According to the DynaMine prediction, ∼70% of Myo16Tail shows high backbone flexibility (S^2^ values < 0.7) and only ∼20% of Myo16Tail has a rigid structure (S^2^ values > 0.8). There is a context-dependent range (10%) where the protein of interest might be able to switch between conformational states or folding can occur toward a more ordered structure upon binding to a partner molecule (49, 50).

As IDRs are enriched in PTM sites, the PTMs of Myo16Tail were analyzed using PhosphoSitePlus (51) (Fig. 2*D*). The prediction showed that several phosphorylation sites might occur in Myo16Tail based on references (11 Ser, 4 Thr, 3 Tyr phosphorylation sites). To assess the relevance of these PTMs, the conservation of predicted Myo16Tail phosphosites was analyzed in the aforementioned multiple sequence alignment (Fig. S1). Three of the predicted phosphorylation sites are located in the NHM motif and shown to be fully conserved from fish to human. Two of them (Tyr^1416^ and Tyr^1441^) were identified to be phosphorylated by Fyn kinase that eventuates the recruitment of the p85 subunit of the PI3K and the activation of the PI3K signaling pathway (17). The rest of phosphosites can be found particularly in the disordered regions, notably 10 of 14 phosphosites are fully conserved, and one of them is weakly similar (Fig. S1). The conservation of phosphosites suggests that phosphorylations in the disordered regions of Myo16Tail might have particular importance in the evolution of vertebrate Myo16.

**Figure 3:**
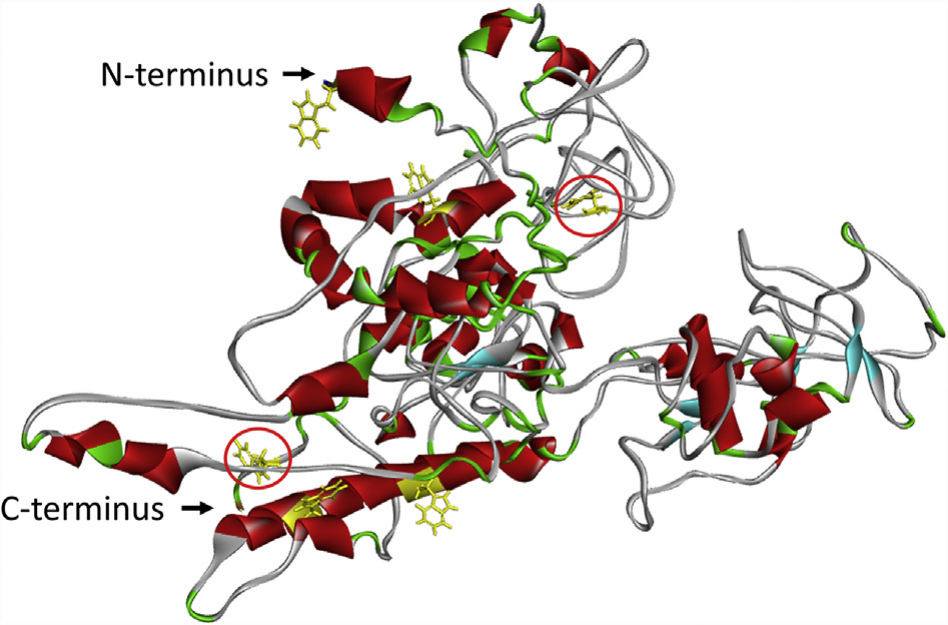
**Myo16Tail displays a molten globule–like structure.** Predicted structural model of Myo16Tail using I-TASSER. Structural elements are represented by different colors: α-helix (*red*), β-sheet (*cyanic blue*), turn (*green*), and disordered structural elements (*gray*). The location of the tryptophan residues is highlighted with *yellow* to visualize their position for the fluorescence spectroscopic assays. Moreover, tryptophans in the predicted disordered segments are featured with *red circles*. Myo16Tail, myosin 16 C-terminal tail; I-TASSER, Iterative Threading Assembly Refinement.

Comprehensively, the sequence-based analysis suggests that Myo16Tail is characterized by an intrinsically disordered structure; however, structural data have not been available yet. For this reason, we created a 3D structural model of Myo16Tail by using Iterative Threading Assembly Refinement (I-TASSER) (52, 53) (Fig. 3). I-TASSER utilizes the Protein Data Bank to identify structural templates by multiple threading approaches. The 3D model of Myo16Tail predicts α-helical, β-sheet, and turn components besides the considerable amount of disordered structural elements. The confidence of the structural model was evaluated by the C-score (confidence-score), which is -1.95 in our model. C-score measures the quality of predicted models in the range of (−5, +2), where the higher the C-score, the more reliable the structural model. The model shows a mixture of secondary structural elements and a significant number of disordered segments, supposing a molten globule–like behavior of Myo16Tail. The molten globule state of a protein is less compact and more flexible and dynamic than a globular fold (54).

### Expression and purification of Myo16Tail

**Figure 4:**
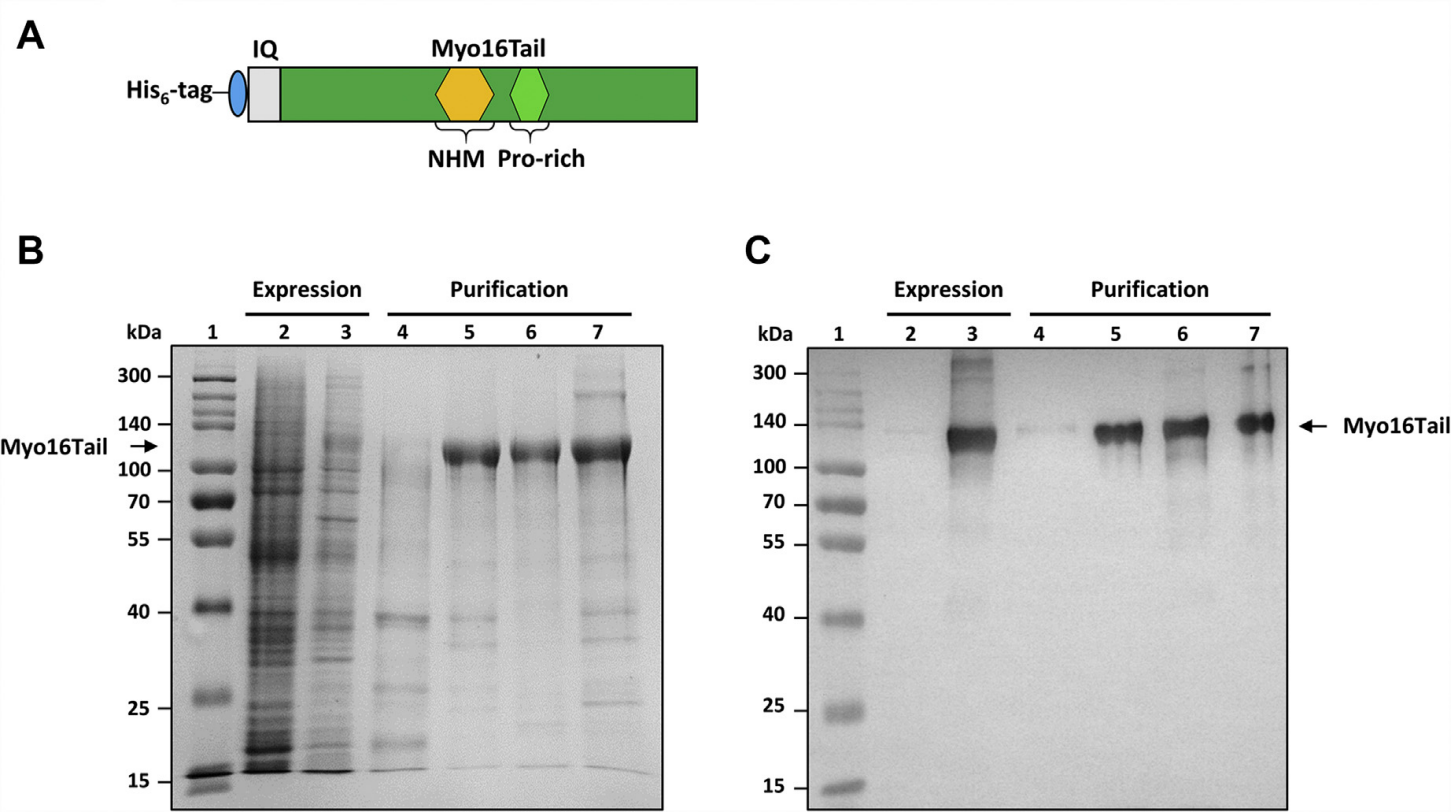
**SDS-PAGE of expressed and purified Myo16Tail.***A*, schematic structure of the His_6_-tagged Myo16Tail construct used in our study. *B*, expression and purification of Myo16Tail. The protein weight standard marker (ProSieve, QuadColor) was applied in lane 1. Total lysate of Sf9 cells as negative control (lane 2) and total lysate of Sf9 cells expressing Myo16Tail (lane 3). Myo16Tail was purified under denaturing conditions using 6 M GuHCl and eluted from the Ni-NTA resin by 8 M urea and decreasing pH; pH 7 is for washing (lane 4), and pH 6 (lane 5) and pH 4 (lane 6) are for elution. At the end of the purification process, Myo16Tail was pooled and concentrated (lane 7). *C*, the presence of Myo16Tail in the expression and purification samples was confirmed by anti-His Western blot analysis. The elution samples of Myo16Tail (pH 6, pH 4, and pooled Myo16Tail) were 5-fold diluted for Western blot to avoid overexposure of chemiluminescence. The membrane was recorded before (molecular weight marker appears) and after developing chemiluminescence. The merged image was used to display the expression and purification of His_6_–Myo16Tail. The samples were derived from the same experiments, and the gel and blot were processed in parallel. GuHCl, guanidine hydrochloride; Myo16Tail, myosin 16 C-terminal tail; Ni-NTA, nickel-nitrilotriacetic acid.

To address experimentally the conformational characteristics of Myo16Tail, we successfully cloned and expressed it in Sf9/baculovirus system (Fig. 4). Because Myo16Tail showed low solubility under native protein preparation conditions, which might be caused by inclusion body formation, a denaturing buffer environment (6 M guanidine hydrochloride [GuHCl], 8 M urea pH 7, 6, and 4) was applied. The denatured, soluble Myo16Tail was purified via a His_6_ affinity tag and renatured by gradually eliminating the denaturant during dialysis. The concentration of routinely purified Myo16Tail was found to be ∼1.7 mg/ml. The solubility of Myo16Tail was assessed by Protein Solubility evaluator II that predicted a value of 0.732, which classifies Myo16Tail as a rather soluble protein (55). The expression and purification results were analyzed and confirmed by SDS-PAGE and Anti-His Western blot (Fig. 4, *B* and *C*). We noted that Myo16Tail showed an anomalous electrophoretic migration (Fig. 4, *B* and *C*). The apparent molecular weight of Myo16Tail seemed to be ∼120 kDa in SDS-PAGE, which is higher than that calculated based on its amino acid sequence (86.47 kDa, ProtParam) (56). This abnormal electrophoretic mobility is a characteristic feature of IDPs, which could result from poor interaction with SDS molecules because of their irregular amino acid composition (28, 39).

### Myo16Tail is functionally active and interacts with Myo16Ank

**Figure 5:**
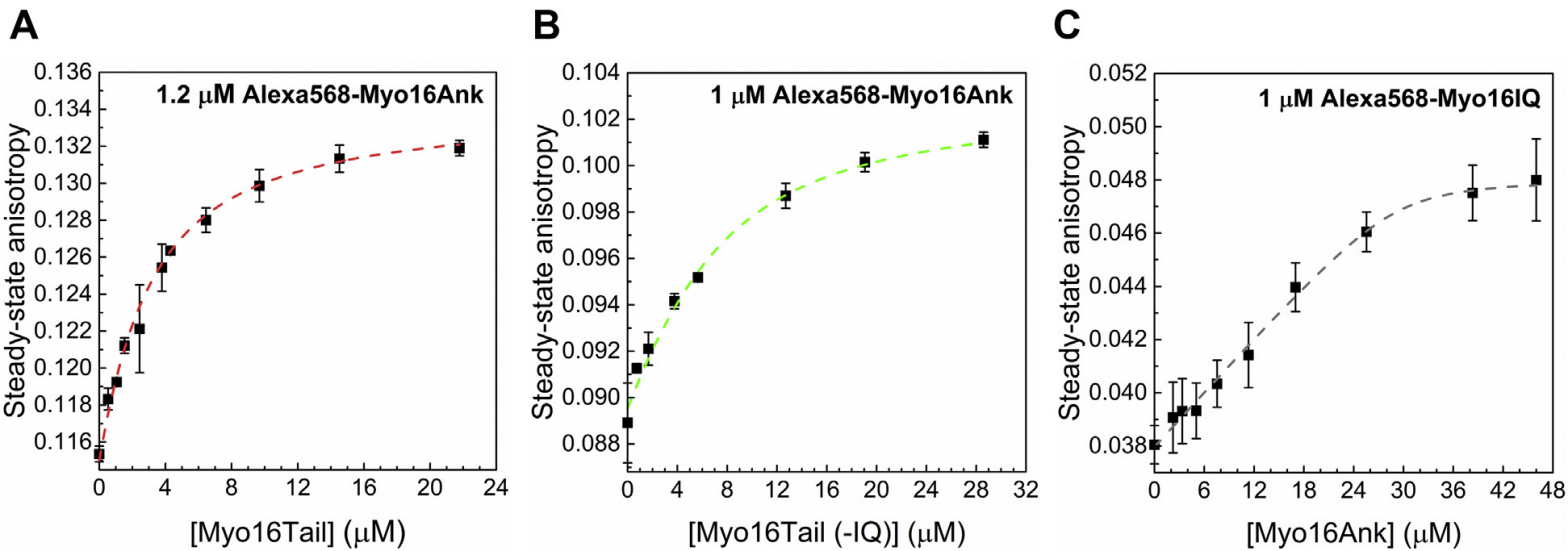
**Myo16Tail interacts with the N-terminal ankyrin domain (Myo16Ank).***A*, steady-state anisotropy of Alexa568–Myo16Ank (1.2 μM) in the absence and presence of an increasing concentration of Myo16Tail. The *dashed line* shows the fit to the data according to Equation 1. The fit of the quadratic binding equation gave a dissociation equilibrium constant of *K*_*D*_ = 2.5 ± 0.2 μM for the Myo16Ank–Tail complex. Mean ± SD (n = 3). *B*, steady-state anisotropy of Alexa568–Myo16Ank (1 μM) in the absence and presence of Myo16Tail (−IQ). The *dashed line* indicates the fit to the data according to Equation 1. The dissociation equilibrium constant resulted in *K*_*D*_ = 5.6 ± 0.02 μM for the Myo16Ank–Tail (−IQ) complex. Mean ± SD (n = 3). *C*, steady-state anisotropy of Alexa568–Myo16IQ in the absence and presence of an increasing concentration of Myo16Ank. The *dashed line* shows the fit to the anisotropy data according to Equation 1, resulting in a dissociation equilibrium constant of *K*_*D*_ = 16.04 ± 2.9 μM for the Myo16IQ–Ank complex. Mean ± SD (n = 3). Myo16Tail, myosin 16 C-terminal tail; Myo16Tail (−IQ), Myo16Tail without the IQ motif.

To study the functional activity of the renatured Myo16Tail, we aimed to characterize its possible binding properties. Backfolding is an intramolecular interaction between the C-terminal tail and the N terminus of myosins that have been described in several classes (myosin IIa, V, VI, VII, X) (57, 58, 59, 60, 61). In general, this mode of autoregulation has a negative impact on the enzymatic activity of the myosin motor. For this reason, we performed a binding assay using steady-state fluorescence anisotropy measurements to test the interaction between Myo16Tails with the N-terminal Myo16Ank region. Anisotropy is a powerful method to investigate the size, shape, dynamics, conformation, and interactions of proteins (62) and was used successfully to monitor protein binding (63, 64, 65, 66, 67). The steady-state anisotropy of fluorescently labeled Alexa568–Myo16Ank (1.2 μM) increased by the addition of an increasing concentration of Myo16Tail as expected for binding interaction. The analysis revealed that the affinity (*K*_*D*_) of Myo16Tail to Myo16Ank is ∼2.5 μM according to Equation 1 (Fig. 5*A*).

To characterize the binding properties of the tail of Myo16 in more detail, we measured the steady-state anisotropy of Alexa568–Myo16Ank (1 μM) in the presence of an increasing concentration of a new recombinant Myo16Tail without the IQ motif, Myo16Tail (−IQ). The fit to the anisotropy data resulted in weaker affinity with ∼5.6 μM dissociation equilibrium constant according to Equation 1 (Fig. 5*B*). Moreover, to investigate the binding contribution of unoccupied IQ motif to Myo16Ank, we measured the steady-state anisotropy of Alexa568-labeled, synthesized rat Myo16IQ in the presence of an increasing concentration of Myo16Ank. Because Myo16IQ is a small peptide (3.6385 kDa) and its motion is probably fast, first we performed time-resolved lifetime measurements with free Alexa568 in the buffer solution and Alexa568–Myo16IQ to confirm the labeling reaction. The average lifetime of free Alexa568 was 2.84 ns, whereas that of Alexa568–Myo16IQ increased to 3.66 ns (Fig. S2). In addition, steady-state fluorescence emission measurements of free Alexa568 dye and Alexa568–Myo16IQ were carried out for further confirmation of the labeling. The maximum wavelength plot showed spectral shift from 596 nm (free Alexa568) to 599 nm (Alexa568–Myo16IQ), which also verifies the labeling of Myo16IQ (Fig. S3). The steady-state anisotropy of Alexa568–Myo16IQ revealed that Myo16Ank is able to interact with Myo16IQ, albeit with much weaker affinity (*K*_*D*_ = ∼16 μM) according to Equation 1 (Fig. 5*C*) to that of both Myo16Ank–Myo16Tail and Myo16Ank–Myo16Tail (−IQ) complexes.

Altogether, on the one hand, our results can confirm the functional activity of the recombinantly produced Myo16Tail and Myo16Tail (−IQ). On the other hand, our anisotropy findings revealed that the tail of Myo16 is dominant in the binding of Myo16Ank; however, the presence of Myo16IQ seems to influence the strength of this interaction. This moderate strength of the interaction of Myo16Tail is relatively common in regulatory functions and consistent with its possible role as a multiple interaction site for PI3K, WRC (17), and Myo16Ank.

### Myo16Tail shows low cooperativity of unfolding

**Figure 6:**
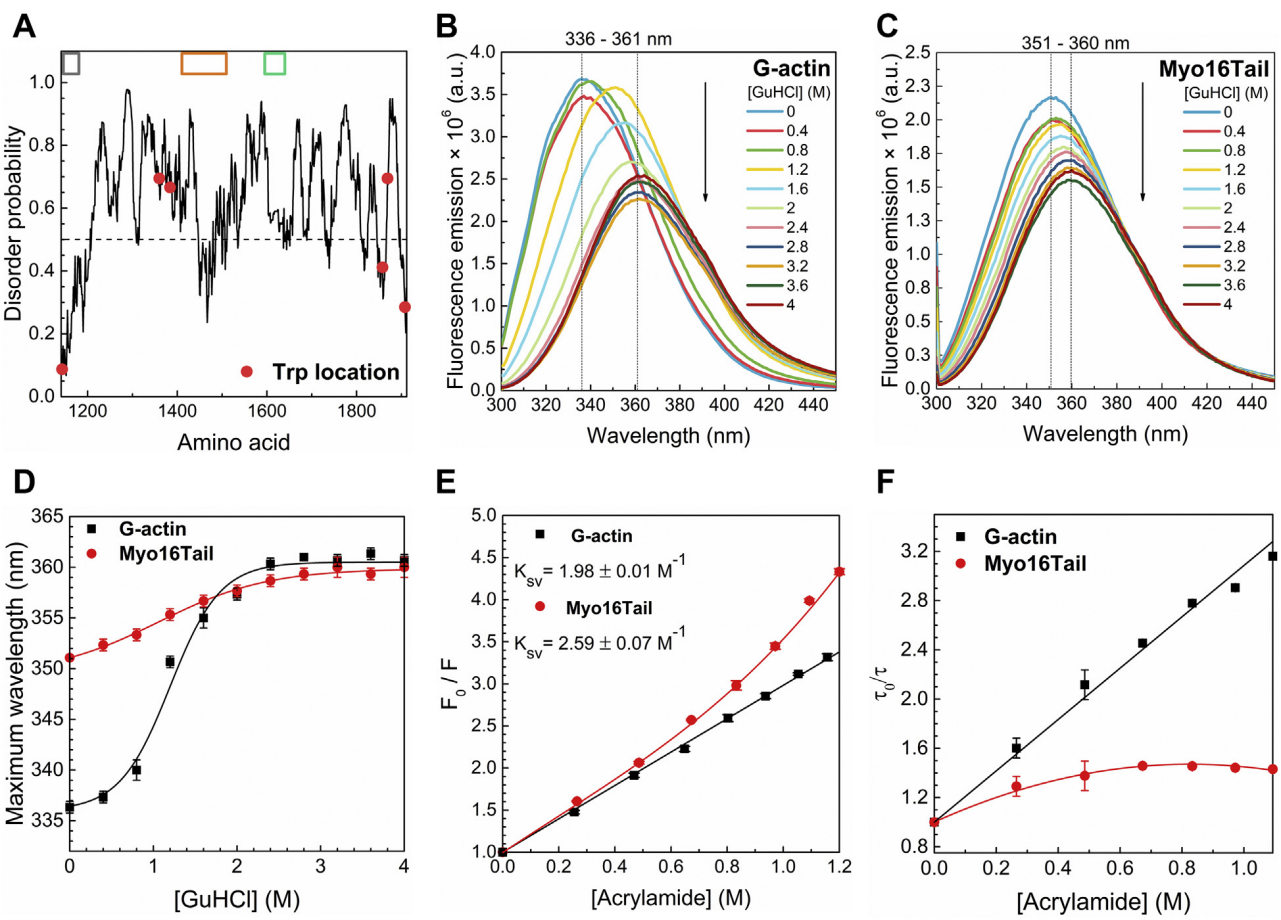
**Conformational transitions followed by intrinsic tryptophan fluorescence emission and tryptophan fluorescence quenching.** Fluorescence emission of tryptophan residues upon the addition of an increasing concentration of the denaturing agent (GuHCl). Tryptophan amino acids were excited at 295 nm, and the emission was recorded between 300 and 450 nm. *A*, the location of tryptophan residues in Myo16Tail. Known regions of Myo16Tail are shown in *gray* (IQ), *orange* (NHM), and *green* (proline-rich) *boxes*. The fluorescence emission of (*B*) G-actin (control) showed a 25-nm red shift in the wavelength corresponding to the emission maximum from 336 to 361 nm, while (*C*) fluorescence emission of Myo16Tail revealed a less-significant red shift of 9 nm from 351 to 360 nm. The *arrows* indicate the increasing GuHCl concentration. *D*, conformational transitions followed by unfolding; emission maximum wavelength (λ_max_) plotted as a function of GuHCl concentration. The sigmoidal fits to the data calculated by using Equation 2 are shown in the corresponding colors. Mean ± SD (n = 3). *E*, the classical Stern–Volmer plot of steady-state fluorescence emission of tryptophans quenched by acrylamide. The fits to the fluorescence quenching data calculated with Equation 4 are shown in the corresponding colors. The data are plotted as the mean ± SD (n = 3), and the K_sv_ values are indicated as the mean ± SD. *F*, the lifetime of tryptophans upon acrylamide quenching using Equation 5. The downward curvature of Myo16Tail indicates the presence of the predominant static component. Fluorescence lifetime values derived from quenching experiments are summarized in Table S1. Mean ± SD (n = 3). G-actin, globular actin; GuHCl, guanidine hydrochloride; Myo16Tail, myosin 16 C-terminal tail; NHM, neuronal tyrosine-phosphorylated adaptor for PI3K homology motif.

Based on the amino acid composition, bioinformatic predictions and structural modeling, we hypothesized the intrinsically disordered nature of Myo16Tail (Figs. 2 and 3). To investigate the structural properties of Myo16Tail experimentally, we performed steady-state fluorescence measurements by using its tryptophan residues as intrinsic probes and ANS fluorescence assays. Myo16Tail contains six tryptophan residues; three of them are located in more ordered segments, whereas the other three are in the predicted disordered regions (Fig. 6*A*). Only one tryptophan residue can be found in the close vicinity of the IQ motif, and the rest of tryptophans are not related to known domains (Fig. 6*A*). In line with this, the position of tryptophan residues in Myo16Tail structural model shows similar distribution (Fig. 3). Tryptophans, as intrinsic fluorophores, are highly sensitive to their local environment (68). According to Reshetnyak et al, tryptophan residues are classified in different classes based on their accessibility (69). The fluorescence emission of tryptophans located in the hydrophobic core of a protein (class S) is characterized by a wavelength corresponding to the maximum fluorescence emission (emission maximum wavelength, λ_max_) of around 320 to 330 nm. The emission maximum of class S tryptophans was at 322.5 ± 4.6 nm (69). Partially buried tryptophans have maximum fluorescence emission at ∼330 to 340 nm characterized by classes I and II, and the average values of tryptophan emission maxima were found to be at 331.0 ± 4.8 and 342.3 ± 3.3 nm, respectively (69). In contrast to class S, when tryptophans are exposed to the solvent, the maximum wavelength is around 350 nm (70), and class III of tryptophan residues was found to have maximum emission at 347.0 ± 3.1 (69) or even can be further red-shifted toward 360 nm (62). To test the results of bioinformatic analysis at first, the tryptophan fluorescence emission of Myo16Tail was measured to reveal the unfolding properties in the presence of increasing concentrations of GuHCl as a denaturing agent. Our presumption was that unfolding of the protein (thus the local environment of tryptophans) should result in a red shift.

Globular actin (G-actin) containing four tryptophans was used as a control, as it has well-defined secondary (71) and tertiary structures (formed by two large domains) (72). The experiments performed on G-actin revealed that in the presence of an increasing amount of GuHCl, the fluorescence emission of tryptophans decreased and the maximum wavelength was red-shifted (25 nm) from 336 to 361 nm (Fig. 6, *B* and *D*). These spectral changes reflect the denaturation of G-actin: as the protein unfolds, the tryptophan side chains are more accessible to the solvent than in the native globular protein structure.

Myo16Tail exhibited a red-shifted maximum wavelength around 350 nm, even in the absence of a denaturant, suggesting that tryptophan side chains are already solvent accessible in the native molecule (Fig. 6, *C* and *D*). The decrease of fluorescence intensity of Myo16Tail upon titration by GuHCl was smaller than that observed for G-actin, as well as the observed red-shift was less pronounced, from 351 to 360 nm (Fig. 6, *C* and *D*). These observations indicate that tryptophans of Myo16Tail are highly accessible, suggesting that they are located in a less-structured, disordered protein matrix. The GuHCl concentration dependence of the maximum wavelength further supported the different conformational characteristics of the two proteins (Fig. 6*D*). The pronounced and steep sigmoid tendency observed for G-actin is indicative of cooperative conformational transitions upon unfolding. In contrast, the trend detected for Myo16Tail suggests modest or the lack of cooperativity (Fig. 6*D*). Cooperativity of protein folding/unfolding denotes the changes of secondary and tertiary interactions, that is, high cooperativity correlates with ordered, globular fold, whereas low cooperativity indicates a disordered structure (73, 74).

To corroborate the above conclusions, tryptophan fluorescence quenching was performed, which is a powerful method to investigate the structural and dynamic properties of proteins (75, 76). Quenching of tryptophan residues of Myo16Tail and G-actin by acrylamide as a neutral quencher was monitored in steady-state fluorescence emission measurements (Fig. 6*E*). The classical Stern–Volmer plot of steady-state fluorescence quenching of tryptophans showed increased K_sv_ values for Myo16Tail (K_sv_ = 2.59 ± 0.07 M^−1^) as compared with that characteristic to G-actin (K_sv_ = 1.98 ± 0.01 M^−1^), suggesting higher tryptophan accessibility and conformational dynamics of Myo16Tail (Fig. 6*E*). As one can observe, the Stern–Volmer plot of Myo16Tail deviates from linear tendency, and a better fit is obtained if we use a quadratic polynomial. This suggests that the quenching mechanism cannot be described only by collisional quenching. An upward curvature was observed in those cases when the fluorophores form a so-called dark complex with the quencher and thus static quenching takes place (77). As the dark complexes cannot be excited, they will not contribute to the measured fluorescence lifetime in the time-resolved measurements. In the case of fluorescence quenching measurements observed by measuring the fluorescence lifetime, the formation of dark complexes will have no effect on the fluorescence lifetime. If only static quenching—and dark complex formation—happens, the τ_0_/τ Stern–Volmer plot will be a flat line. To check the contribution of static quenching, we performed acrylamide quenching experiments by measuring the fluorescence lifetimes using time-correlated single-photon counting (TCSPC) (Fig. 6*F*). As one can see in Figure 6*F*, the Stern–Volmer plot of G-actin is linear, and we can conclude that the quenching is collisional. Contrary to the quenching measurements on G-actin, the Stern–Volmer plot of measurements on Myo16Tail shows a moderate increase until the addition of 0.5 M acrylamide; after that, it is relatively flat (Fig. 6*F*). This suggests that in the case of Myo16Tail, there is a significant static quenching part aside from the collisional quenching. Observing the average fluorescence lifetime of tryptophans of Myo16Tail, one can see that the lifetime drops significantly—from 4.1 ns to 3.0 ns—by adding ∼0.5 M acrylamide. Above this concentration, the increasing amount of acrylamide has only a minor effect on the average lifetime, which levels of around 2.8 ns. In comparison, the quencher has a stronger effect in the case of G-actin: as one can observe, the average lifetime of tryptophans decreases linearly with an increasing quencher concentration (Table S1). In the time-resolved quenching measurements, the increasing quencher concentration results in decreasing fluorescence lifetime of Myo16Tail, which means that above ∼0.5 M, the acrylamide forms a dark complex with the tryptophans. The possible explanation of this finding is that the solvent-exposed tryptophans are quenched efficiently by acrylamide.

To study the structural behavior of Myo16Tail in more detail, 1-anilino-naphthalene-8 sulfonic acid (ANS) fluorescence measurements were performed. ANS is a hydrophobic fluorescent probe that is used to reveal the hydrophobic sites and characterize the molten globule conformation of proteins (78). The change of fluorescence intensity and spectral distribution by increasing GuHCl concentration reflects alterations in the microenvironment of ANS from a hydrophobic (well-structured) to a polar environment (solvent-exposed–less structured). ANS does not bind to well-ordered proteins, or to totally unfolded ones, but it displays maximum intensity when molten globule conformation can occur (78).

**Figure 7:**
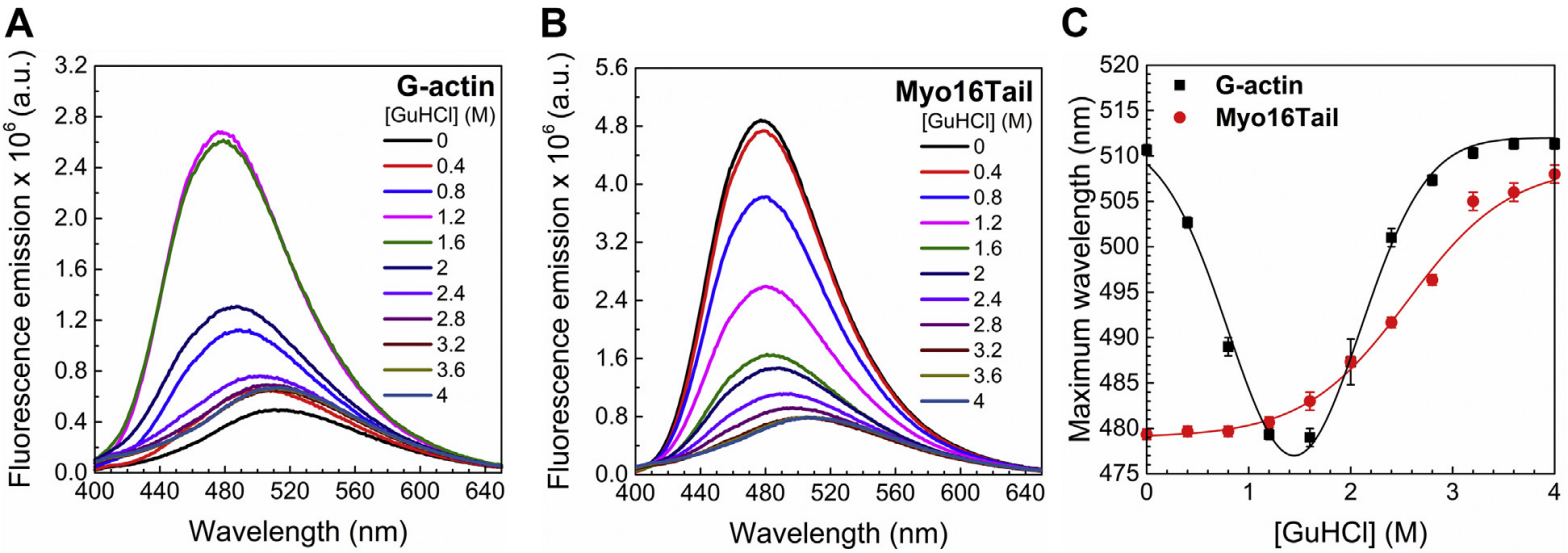
**Hydrophobic core accessibility monitored by ANS fluorescence upon GuHCl unfolding.** ANS was excited at 360 nm, and the emission was recorded between 400 and 650 nm. *A*, ANS fluorescence of G-actin (control) revealed the highest ANS intensity between 1 and 2 M GuHCl concentration, which is indicative of hydrophobic (nonpolar) to solvent-exposed (polar) microenvironment change. *B*, ANS fluorescence of Myo16Tail showed the maximal ANS fluorescence intensity in the absence of GuHCl and constantly decreased upon unfolding. *C*, conformational transitions of the emission maximum wavelength of ANS fluorescence as a function of GuHCl concentration. G-actin showed cooperative unfolding by using Gaussian fit to the data (Equation 3.), whereas Myo16Tail resulted in low cooperative unfolding process. The sigmoidal fit to the data of Myo16Tail was derived by using Equation 2. Mean ± SD (n = 3). ANS, 1-anilino-naphthalene-8 sulfonic acid; G-actin, globular actin; GuHCl, guanidine hydrochloride; Myo16Tail, myosin 16 C-terminal tail.

In the case of G-actin (control) in the absence of GuHCl, the ANS fluorescence intensity was relatively low, which constantly increased and reached a maximum between 1 and 2 M GuHCl, suggesting partial denaturation and molten globule transition of G-actin (Fig. 7*A*). Above this range of GuHCl concentration, the fluorescence intensity decreased toward the initial fluorescence emission values showing a cooperative unfolding of G-actin (Fig. 7*C*).

Myo16Tail showed a relatively high ANS fluorescence intensity already in the absence of GuHCl that decreased upon GuHCl addition in a sigmoidal manner with a fewer steep transitions than that observed for G-actin (Fig. 7, *B* and *C*). Importantly, the highest intensity and wavelength maximum of ANS fluorescence of Myo16Tail at low GuHCl (∼480 nm) is in accordance with the maximum values of G-actin in the range of 1 to 2 M GuHCl (∼480 nm), when the latter one is in molten globule conformation (Fig. 7*C*). Our ANS fluorescence results corroborate that Myo16Tail might have molten globule conformation under native conditions.

### Conformation dynamics of Myo16Tail

**Figure 8:**
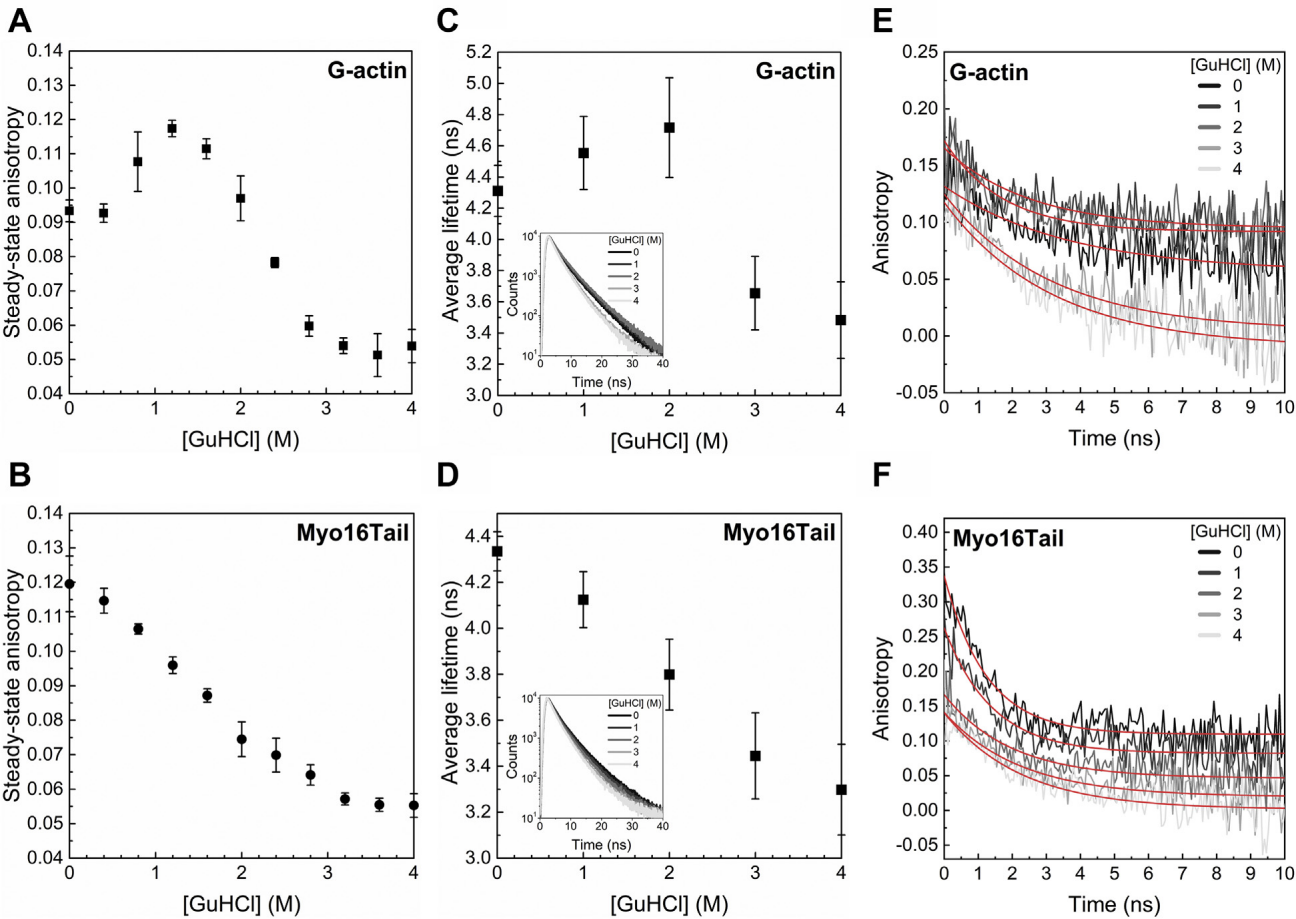
**Structural dynamic measurements using tryptophan fluorescence anisotropy.** Steady-state anisotropy of (*A*) G-actin (control) and (*B*) Myo16Tail as a function of GuHCl concentration. Mean ± SD (n = 3). Time-correlated single-photon counting of tryptophan residues by adding GuHCl. The excitation wavelength was 295 nm, and the emission was monitored at 350 nm, using slits of 5 to 5 nm. Time-resolved lifetime plot of unfolding of (*C*) G-actin (control) and (*D*) Myo16Tail; the raw lifetime decays are indicated as insets. The lifetime data (mean ± SD, n = 3) were derived according to Equation 6 and shown in Table S2. Anisotropy decay of G-actin (control) (*E*) and Myo16Tail (*F*) upon denaturation. The *red lines* show the fit to the data according to Equation 7. The derived rotational correlation (θ) data (mean ± SD, n = 3) are shown in Table S2. G-actin, globular actin; Myo16Tail, myosin 16 C-terminal tail; GuHCl, guanidine hydrochloride.

To further assess the conformational properties of Myo16Tail, steady-state and time-resolved anisotropy measurements were carried out (Fig. 8). At first, the steady-state tryptophan fluorescence anisotropy of G-actin and Myo16Tail was compared upon chemical denaturation induced by GuHCl (Fig. 8, *A* and *B*). In the case of G-actin (control), a two-step process could be observed, in agreement with previous reports (79). First, at low GuHCl concentrations, the transition of G-actin from native to inactivated state can be revealed by the increase in fluorescence anisotropy. Subsequently, a steep decrease occurs indicating the transformation of inactivated G-actin into an unfolded state (79) (Fig. 8*A*). A less-complex response was detected for Myo16Tail upon addition of GuHCl; also, the low steepness of the transition curve suggests low cooperativity in unfolding (80) (Fig. 8*B*), in agreement with the fluorescence emission measurements and with the ANS fluorescence findings (Fig. 6, *B*–*D*, Fig. 7, *B* and *C*).

TCSPC data showed that the decrease in tryptophan lifetime correlates upon denaturation by an increasing concentration of GuHCl (1, 2, 3, 4 M) (Fig. 8, *C* and *D*). The average lifetime of tryptophan residues of G-actin as a function of the denaturant concentration (Fig. 8*C*; Table S2) showed similar tendency to that of steady-state anisotropy data (Fig. 8*A*). For Myo16Tail, the average lifetime of tryptophans (Fig. 8*D*) revealed a similar, slightly sigmoidal decline upon chemical denaturation as we observed in steady-state anisotropy measurements (Fig. 8*B*).

The anisotropy decay of G-actin with a molecular mass of 42 kDa showed similar tendency in tryptophan rotation to that of the lifetime decay (Fig. 8*E*; Table S2). The tendency in the change of G-actin lifetime and anisotropy decay can be explained by the two consecutive conformational transitions during unfolding (79). The anisotropy decay data measured in the case of G-actin are in correspondence with previously described findings (81), starting with a rotational correlation time of ∼26 ns and decreasing with increasing GuHCl concentrations. The reported values show the rotation of the whole protein (Table S2). Decreasing rotational correlation times reflect the increased mobility of the segments having the tryptophans, meaning that upon denaturation, these parts of the protein became more solvent-exposed, therefore more mobile. In the case of Myo16Tail in the absence of GuHCl, one can observe a very different anisotropy decay that can be fit with two exponentials, a faster ∼1 ns and a longer ∼33 ns phase (Fig. 8*F*; Table S2). The fast phase does not appear in the case of G-actin, and it can be assigned as the standalone rotation of tryptophan residues. The longer phase detected for Myo16Tail is practically equal to the expected value for a protein using the experimental formula by Visser (82) with the corresponding molecular weight (∼86.5 kDa). This result shows that in Myo16Tail, there is an ensemble of tryptophans, which are at least partially unburied and they are more solvent exposed than in the case of G-actin. The rotation of these solvent-exposed tryptophans is less restricted, which is the reason of the presence of the ∼1 ns component in the rotational correlation times.

These findings agree with the correlation between the locations of tryptophan residues in the predicted structural model (Fig. 3) and the disorder probability of the corresponding regions (Fig. 6*A*). When titrating Myo16Tail by increasing the GuHCl concentration, one can observe a slight decrease of both components, suggesting that the segments having the tryptophans became more mobile upon denaturation; the increase in the mobility however is not as obvious as in the case of G-actin. Altogether, both the steady-state and time-correlated fluorescence results indicate that Myo16Tail can undergo slight conformational transitions upon unfolding, suggesting that the disordered regions are associated with some ordered structural elements.

### Secondary structure analysis of Myo16Tail by CD spectroscopy reveals structured regions

**Figure 9:**
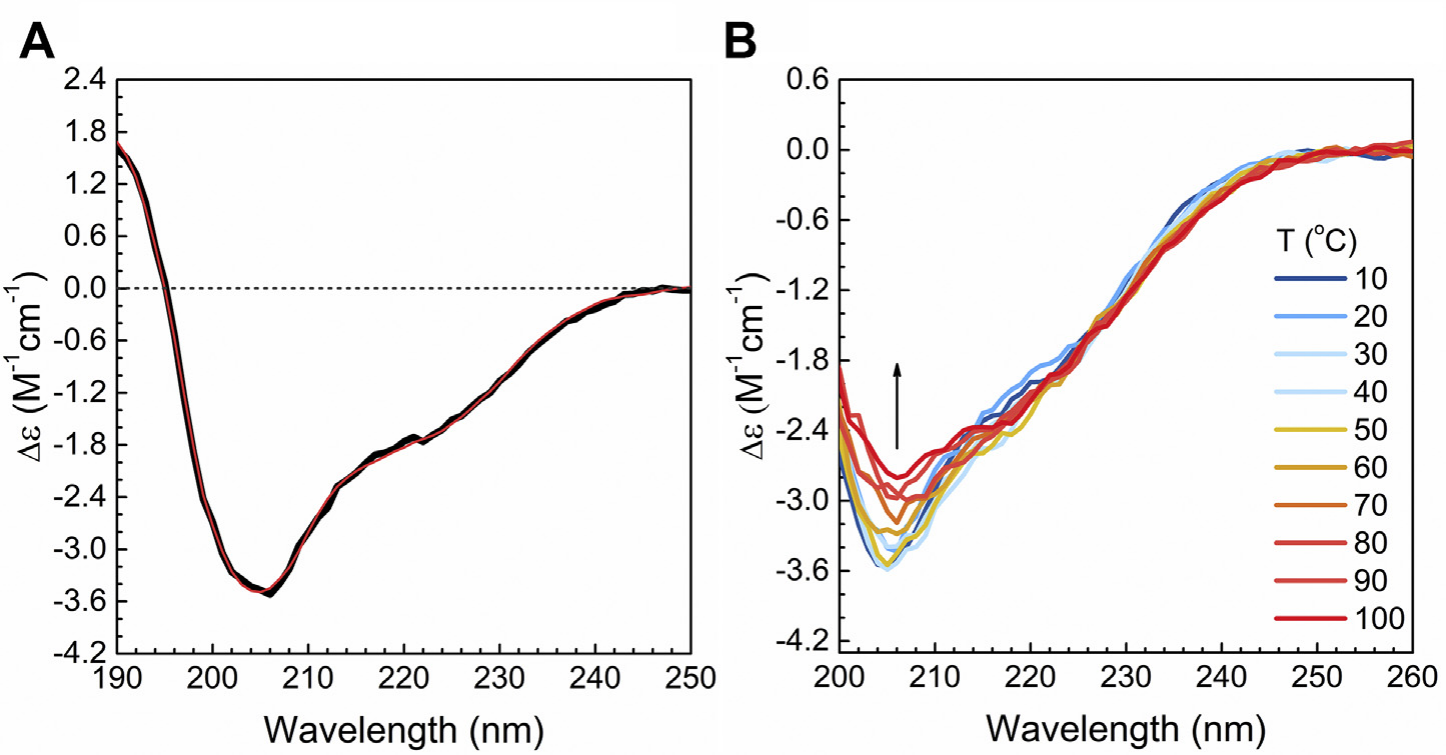
**Secondary structure analysis of Myo16Tail using CD spectroscopy.***A*, far-UV CD spectrum of Myo16Tail (12.7 μM (1.1 mg/ml)) in the solution (*black*) and fit using the BeStSel method (85, 86) (*red*). *B*, thermal denaturation of Myo16Tail (1.15 μM (0.1 mg/ml)). The CD spectra were recorded at 10 °C steps in the 10 to 100 °C range. The *arrow* indicates the direction of changes. BeStSel, Beta Structure Selection; Myo16Tail, myosin 16 C-terminal tail.

To study the secondary structure of Myo16Tail, CD spectroscopy measurements were performed, which is a widely used method for structural characterization of proteins in solutions (83) (Fig. 9). The CD spectrum of Myo16Tail in the far-UV region revealed the minimum at 205 nm, the positive maximum at 190 nm, and a significant signal in a wide, 215 to 225 nm region (Fig. 9*A*). This suggests that the protein contains both α-helical and β-structured elements. On the other hand, the large minimum at 205 nm and the relatively weak positive maximum around 190 nm indicate the presence of a significant amount of disordered structure as well. The CD spectroscopic analysis of G-actin (control) that was described and published earlier revealed two major negative minima in the far-UV region at 211 nm and 221 nm, suggesting a significantly higher amount of α-helical and β-structured elements than what we detected in Myo16Tail (84).

To evaluate the secondary structure content of Myo16Tail, Beta Structure Selection (BeStSel) method (85, 86) was used as a deconvolution algorithm to assess CD data. BeStSel is a novel method to distinguish between the α-helical content and β-sheet structural elements and also to differentiate parallel and antiparallel β-sheets, providing an improved secondary structure estimation for a wide range of proteins. Antiparallel β-sheets can be divided into subclasses such as anti1 (left-twisted), anti2 (relaxed), and anti3 (right-twisted) β elements.

BeStSel analysis showed that the secondary structure content of Myo16Tail appeared to be due to 19.5% α-helix and 21.3% β-sheet, where the contributions of antiparallel β subclasses are 0.0% anti1 (left-twisted), 5.4% anti2 (relaxed), and 15.9% anti3 (right-twisted). Further structures in form of 15.2% turn and 44.0% “others” are present, the latter accounts mainly for the disordered content. Of note, besides our functional analysis, the secondary structure results also confirm the appropriate renaturation of Myo16Tail during protein purification. CD spectroscopy results revealed that Myo16Tail contains ordered secondary structure elements. We hypothesize that the helical polyproline II type recognition motifs in the C-terminal and Pro-rich regions (87) are also formed. These findings are in line with the disorder prediction, in which some segments are predicted to be ordered (Fig. 2*A*). However, the sequence of IQ motif predicted to be ordered accounts for only 3.8% of the sequence of the whole construct, suggesting that IQ might have only minor contribution to the secondary structure components and helical and sheet structural elements predominantly account for the C-terminal part of Myo16Tail. Moreover, it is important to emphasize the presence of a high number of disordered regions. Altogether, the turn and disordered structure make up at least 60% in Myo16Tail, which is consistent with the primary amino acid sequence–based bioinformatic findings. It is important to note that in highly disordered proteins, some part of the disordered content might be counted as anti3 component by BeStSel (86), which is the case here, suggesting an even higher level of disorder. Similar trend in CD spectroscopy data was observed for Golgi Reassembly and Stacking Proteins, suggesting a natively unstructured, molten globule–like behavior (88). The mean residue ellipticity of far-UV CD signals of Myo16Tail was analyzed on a double-wavelength plot, [θ]_222_ against [θ]_200,_ provided by Uversky and Fink (89) to classify Myo16Tail as a molten globule or pre-molten globule conformation (Fig. S4). Based on the plot, Myo16Tail can be found between the molten and pre-molten globule populations.

To further study the structural properties of Myo16Tail by CD, thermal denaturation experiments were carried out by heating up the protein from 10 °C to 100 °C and recording the spectra at 10 °C steps (Fig. 9*B*). The CD spectra gradually changed upon increasing the temperature, although Myo16Tail showed only minor spectral changes without a well-defined unfolding transition. At higher temperatures, some precipitation of the protein was observable; however, even with precipitation, there were only minor spectral changes. The secondary structure analysis showed a ∼7% decrease in the α-helix content and ∼5% increase in the disordered content. The β-structure content is increased by ∼4%, which could be the result of partial aggregation of the protein at high temperatures. The thermal behavior of Myo16Tail showing low cooperativity detected by CD measurements supports the idea of lacking a stable globular fold and is characteristic of a molten globule–like state (90). In conclusion, CD spectroscopy analysis indicates the presence of both disordered and structured regions in Myo16Tail, supporting our conclusions from fluorescence spectroscopic measurements according to which the tail of Myo16 might be in a molten globule–like conformation.

## Discussion

The unconventional Myo16 is a relatively poorly characterized member of the myosin superfamily. The tail in Myo16b isoform is a unique extension involved in the neuronal PI3K signaling pathway (8, 17) and supposed to have intrinsically disordered structure (15). However, its detailed biological functions and structural features remained unclear. Here, a combination of bioinformatic and spectroscopic methods was used to investigate the functional activity, structural properties, and behavior of Myo16Tail. The bioinformatic analysis showed that Myo16Tail has a considerable number of disordered regions in its structure. The experimental data by tryptophan and ANS fluorescence emission, steady-state and time-resolved anisotropy, and CD confirmed the bioinformatic predictions and provided insights into the structural characteristics of Myo16Tail.

Myo16Tail was assessed by five disorder predictors and by DynaMine protein flexibility tool (Fig. 2). The NHM, the Pro-rich motif, and the C terminus overall appear to be disordered in all predictions, and only the IQ motif has ordered structure as we expected (47). Moreover, certain parts of the NHM motif and the C terminus of Myo16Tail are predicted to be close to the ordered–disordered threshold, suggesting that these regions might be able to fold into more ordered structure, for example, upon binding to partner proteins (Fig. 2*A*). The disorder predictions correlate well with the DynaMine results (Fig. 2*C*). The IQ motif displays a rigid protein backbone related to its ordered secondary structure content. In contrast, the NHM, the Pro-rich motif, and the C terminus are predicted to fall mainly in the flexible range associated with disorder. Some portions of these regions are in the context-dependent range in agreement with the disorder prediction, suggesting contextual folding. Intrinsic disorder is associated with conformational flexibility, which correlates with PTMs, such as phosphorylation. Myo16Tail is regulated by phosphorylation in the neuronal PI3K signaling pathway (17). Myo16Tail sequence assessment by PhosphoSitePlus predicts conserved phosphorylation sites in the NHM and the disordered regions, which is consistent with the abundance of phosphosites in disordered and flexible protein structure (Fig. 2*D*). The multiple sequence alignment analysis suggests high conservation of the disordered segments between Myo16Tail amino acid sequences from different vertebrate species (Fig. S1), indicating that our observations on rat Myo16Tail can have relevance, particularly in human Myo16 associated with neurodegenerative diseases (21, 22, 23). The structural model supports the intrinsically disordered nature of Myo16Tail (Fig. 3).

The first experimental indication of the disordered structural characteristics of Myo16Tail is its anomalous migration showing higher apparent molecular mass as revealed by SDS-PAGE, which was confirmed by anti-His Western blot (Fig. 4, *B* and *C*). The unusual SDS-PAGE mobility as a characteristic feature of IDPs (28, 39) supports that Myo16Tail or parts of it are intrinsically disordered. Steady-state fluorescence anisotropy revealed that both Myo16Tail and Myo16Tail (−IQ) bind the N-terminal Myo16Ank with a *K*_*D*_ of ∼2.5 μM and ∼5.6 μM in a similarly moderate range of affinity, respectively (Fig. 5, *A* and *B*), confirming the functional activity and a possible role of Myo16Tail. In our assumption, Myo16Tail may play a role in regulation by backfolding to the N terminus of Myo16. In addition, GuHCl denaturation of Myo16Tail followed by tryptophan fluorescence emission revealed that it lacks the globular fold of well-structured proteins. The relatively small shift in the tryptophan emission spectrum toward red wavelengths correlates with the solvent-exposed environment of tryptophans, suggesting low conformational complexity (Fig. 6*C*). Moreover, the emission maximum wavelength profile of Myo16Tail clearly showed a shallow sigmoidal transition, implying the presence of ordered protein segments (Fig. 6*D*). Fluorescence quenching results support that tryptophans of Myo16Tail are highly accessible, which is indicative of a dynamic and flexible structural behavior (Fig. 6, *E* and *F*). ANS fluorescence measurements corroborate that the hydrophobic core of Myo16Tail is solvent-exposed, having the highest intensity in the absence of a denaturant (Fig. 7*B*). Furthermore, the wavelength maxima of ANS emission change in a poorly cooperative way, suggesting molten globule conformation of Myo16Tail (Fig. 7*C*). The steady-state and time-correlated fluorescence anisotropy results agree well with the dual structural nature of Myo16Tail (Fig. 8). Furthermore, CD revealed α-/β-type secondary structural elements besides the considerable amount of disordered content in Myo16Tail (Fig. 9*A*). The analysis of the mean residue ellipticity of Myo16Tail CD signals on the double-wavelength ([θ]_222_ against [θ]_200_) plot (89) showed the location between the population of molten globule and pre-molten globule conformations (Fig. S4). Moreover, thermal denaturation experiments indicate minor conformational changes and the lack of cooperative unfolding behavior, supporting the dominantly unstructured nature of Myo16Tail (Fig. 9*B*).

Altogether, our results suggest that Myo16Tail might possess a high number of IDRs besides ordered structural elements. Moreover, the shallow, sigmoid cooperative unfolding of Myo16Tail indicates molten globule–like behavior (Figs. 6*D*, 7*C*, and 8, *B* and *C*) rather than pre-molten globule conformation because denaturant-induced unfolding of pre-molten globules or natively unfolded coils shows noncooperative and unfeatured linear changes (80), which was not observed in our experiments. In line with this, the structural model of Myo16Tail corroborates our experimental findings.

Therefore, we propose that Myo16Tail might act as a less compact, dynamic molten globule (54), rather than a pre-molten globule, and may function as a flexible display site (91). Proving the presence of IDRs may help explain how Myo16 behaves during interactions with binding partners (Myo16Tail interacts with PI3K and WAVE1, Myo16Ank), in phosphorylation process (Myo16Tail phosphorylated by Fyn) or signaling pathways playing crucial role in regulation (17). The plasticity of disordered proteins is an evolutional advantage as a response to contextual change due to binding and environmental fluctuations (33). These disorder-associated functions are hypothesized to be evolutionary directed and crucial for certain functions (36, 37). As examples, both regulation by signaling and neurodegenerative diseases are associated with proteins possessing intrinsically disordered structure (92). From this aspect, it is of note that the genetic alterations of *MYO16* are implicated in neurodegenerative disorders (21, 22, 23).

## Experimental procedures

### Bioinformatics

Disordered probability prediction for Myo16Tail was performed using VLXT (39, 40), VL3-BA (41), VSL2b (42), RONN (43), and IUPred servers (44, 45, 46). Structural flexibility was analyzed by DynaMine server (49, 50). Phosphorylation site prediction was assessed using PhosphoSitePlus (51). The physical and chemical parameters of Myo16Tail were calculated using ExPASy ProtParam tool (56), and the protein solubility was evaluated with Protein Solubility evaluator II tool (55). Multiple sequence alignment of Myo16Tail was performed by using Clustal X (48). The 3D structural model was created by using I-TASSER (52, 53).

### Protein expression and purification

The DNA sequence of Myo16Tail (Gene Bank accession number: 192253, *R. norvegicus,* amino acid residues: 1146–1912) (Figs. 1 and 4*A*) containing the IQ motif was optimized by GenScript for baculovirus/Sf9 system. Myo16Tail was cloned into pFastBac plasmid (Thermo Fisher Scientific) containing a His_6_ affinity tag at the N terminus of the construct. Recombinant His_6_–Myo16Tail was expressed in Insectagro Sf9, Serum-Free/Protein-Free 1× medium (Corning) containing 100 μg/ml antibiotics-antimycotics (Biowest). After 2 days of expression, Sf9 cells were harvested and the cell pellet was frozen in liquid nitrogen for further utilization. All steps of Myo16Tail purification were performed in sodium phosphate (Na_3_PO_4_) buffer (an appropriate mixture of 0.2 M Na_2_HPO_4_ and 0.2 M NaH_2_PO_4_) (93) at 4 °C. The cell pellet was extracted in Na_3_PO_4_ lysis buffer (50 mM Na_3_PO_4_ (pH 8.0), 500 mM NaCl, 10 mM β-mercaptoethanol [BME], 0.2 mM PMSF, 1% Triton X-100). After mechanical homogenization and sonication (BANDELIN SONOPULS GM 3100; MS 73 Titanium microtip, 4 × 1 min, rest 1 min, amplitude 80%, frequency 0.4 s) of the cell lysate, 20 μg/ml DNase (PanReac, AppliChem) and Protein Inhibitor Cocktail (P8465, Sigma-Aldrich) were added and the solution was stirred for 1 h. After ultracentrifugation of the cell lysate (Hitachi; 20,000*g*, 20 min, 4 °C) Myo16Tail sedimented in the pellet. To keep Myo16Tail in a soluble form, 6 M GuHCl was added and the pellet was dissolved using mechanical homogenization. The second ultracentrifugation (Hitachi, 20,000*g*, 20 min, 4 °C) was followed by the addition of 20 μg/ml RNase A (Thermo Fisher Scientific) to the supernatant for eliminating free RNA. Afterward, the His_6_–Myo16Tail was incubated overnight with Ni-NTA resin (Protino Ni-NTA Agarose) in the presence of 10 mM BME. The resin was loaded into the column, and it was washed with the washing buffer (8 M urea, 50 mM Na_3_PO_4_, 500 mM NaCl, 10 mM BME) at pH 7. His_6_–Myo16Tail was eluted with the elution buffer (8 M urea, 50 mM Na_3_PO_4_, 300 mM NaCl, 10 mM BME) at pH 6 and pH 4, and the eluted fractions were collected. The samples were run by SDS-PAGE, the peak fractions were pooled and dialyzed first against Na_3_PO_4_ buffer (50 mM Na_3_PO_4_ (pH 8.0), 200 mM NaCl, 5 mM BME) containing 4 M urea and afterward against 50 mM Na_3_PO_4_ buffer containing 100 mM NaCl, 2.5% glycerol, 1% sucrose, and 5 mM BME, and finally against 50 mM Na_3_PO_4_ buffer containing 100 mM NaCl and 5 mM BME. Myo16Tail was concentrated (Amicon Ultra 50-kDa cutoff, Sigma-Aldrich), and after a clarifying centrifugation (Beckman; 1000*g*, 2 min, 4 °C), was stored on ice until use. For the binding assays, an additional recombinant Myo16Tail was generated. The tail of Myo16 was cloned into the pFastBac vector without the IQ motif (Myo16Tail (−IQ), 1176–1912 amino acids) and expressed in the baculovirus/Sf9 system. The recombinant His_6_–Myo16Tail (−IQ) was successfully purified by the abovementioned denaturing procedure. The absorbance of Myo16Tail and Myo16Tail (−IQ) samples were determined at 280 nm by using the Jasco V-660 UV-VIS spectrophotometer (JASCO), and the protein concentrations were calculated using the molar extinction coefficients (ε = 58.330 M^−1^ cm^−1^, 52.830 M^−1^ cm^−1^), respectively. The N-terminal ankyrin domain of Myo16 (Myo16Ank) was purified as described earlier (15). Actin was purified according to standard protocols and stored in G-buffer (4 mM Tris-HCl [pH 7.8], 0.2 mM ATP, 0.1 mM CaCl_2_, 0.5 mM BME) on ice (66). Myo16IQ (*R. norvegicus,* amino acid residues: 1146–1175, MW: 3.6385 kDa, determined by mass spectrometry) was synthesized by the company (GenScript) in a final concentration of 4.9 mg/ml. The lyophilized Myo16IQ was dissolved first in 1 ml of ultrapure water, and then, the solution composition was readjusted with the concentrated buffer to get the final buffer conditions in 50 mM Na_3_PO_4_ buffer containing 100 mM NaCl and 5 mM BME.

### Western blot analysis

Samples were separated on 10% SDS-polyacrylamide gel and then transferred to a nitrocellulose membrane with Trans-blot Turbo Transfer System (Bio-Rad). Nonspecific binding sites were blocked with 3% nonfat milk in Tris buffered saline containing 0.5% Tween-20. During immunodetection, the membrane was incubated with anti-His antibody (Sigma-Aldrich mouse monoclonal IgG SAB1305538, dilution 1:3000) for 1.5 h followed by horseradish peroxidase–conjugated rabbit anti-mouse secondary antibody (Millipore AP160P, dilution 1:10,000) for 45 min. The immunoreactive bands were detected by Luminata Crescendo Western HRP Substrate (Merck) and imaged with the Multi-Genius Bio imaging System (Syngene).

### Protein labeling

Myo16Ank and Myo16IQ were labeled by Alexa Fluor C5 568 maleimide (Alexa568, Invitrogen) according to the following protocol. Alexa568 was added at 10-fold molar excess to the protein solution while stirring in the BME-free buffer (50 mM Na_3_PO_4_ buffer containing 100 mM NaCl). The sample was incubated overnight at 4 °C, and the reaction was terminated by adding 10 mM BME. The solution was dialyzed against BME containing the buffer overnight at 4 °C to remove the unbound dye. The protein sample concentration was corrected with the absorption of the dye at 280 nm.

### Steady-state fluorescence anisotropy

The steady-state anisotropy of Alexa568–Myo16Ank (1 μM and 1.2 μM) and Alexa568–Myo16IQ (1 μM) was measured to study the interaction of Myo16Tail and Myo16Tail (−IQ) with Myo16Ank and Myo16Ank with Myo16IQ. The fluorescence anisotropy measurements of tryptophan side chains were performed on the samples by adding the aforementioned series of GuHCl concentration. The measurements were performed using HORIBA Jobin Yvon Fluorolog 3.22 spectrofluorometer (HORIBA Scientific) (tryptophan, λ_ex_ = 295 nm, λ_em_ = 350 nm; Alexa568–Myo16Ank and Alexa568–Myo16IQ, λ_ex_ = 578 nm, λ_em_ = 601 nm). We analyzed the data with Origin 2020 software (OriginLab). The anisotropy data were evaluated with the quadratic binding equation (by using Equation 1), where A_0_ and T_0_ are the total Myo16Ank, Myo16IQ and Myo16Tail, Myo16Ank concentrations, respectively, r_A_ is the steady-state anisotropy of Alexa568–Myo16Ank or Alexa568–Myo16IQ, r_AT_ is the steady-state anisotropy of Alexa568–Myo16Ank or Alexa568–Myo16IQ at a saturating amount of Myo16Tail or Myo16Ank concentration, respectively, and *K*_*D*_ is the dissociation equilibrium constant of the Myo16Ank–Tail or Myo16IQ–Ank complex:

(1) $$\frac{r-r_{A}}{r_{AT}-r_{A}}=\frac{A_{0}+T_{0}+K_{D}-\sqrt{{\left(A_{0}+T_{0}+K_{D}\right)}^{2}-4\cdot A_{0}\cdot T_{0}}}{2}$$

### Steady-state fluorescence emission measurements

Fluorescence emission of tryptophan side-chains and ANS was monitored by HORIBA Jobin Yvon Fluorolog 3.22 spectrofluorometer using 5 to 5 μM of G-actin and Myo16Tail. Tryptophans were excited at 295 nm, and the emission spectra were recorded between 300 and 450 nm, while ANS (250 μM) was excited at 360 nm, and the emission was monitored from 400 to 650 nm using 2.5 to 2.5 nm slit in the absence and presence of increasing concentrations of GuHCl (0, 0.4, 0.8, 1.2, 1.6, 2, 2.4, 2.8, 3.2, 3.6, 4 M) at 20 °C. The tryptophan fluorescence emission spectra of proteins were corrected by subtracting the GuHCl background intensity. To evaluate the cooperativity of unfolding, we used Origin 2020 software and applied a sigmoidal function (by using Equation 2) (94, 95) to fit the maximum wavelength data of tryptophan fluorescence emission (Fig. 6*D*), where λ_N_ and λ_U_ are the maximum wavelength values of the native and unfolded protein, respectively, D is the concentration of the denaturant, and a is inversely proportional to the slope of the transition curve at D.

(2) $$\lambda =\lambda_{N}+\frac{\left(\lambda_{U}-\lambda_{N}\right)}{1+\exp\left(\frac{D-x}{a}\right)}$$

In the case of the ANS fluorescence of G-actin control, the Gaussian function was fitted by using Equation 3 (Fig. 7*C*), where A is the area, y_0_ is the base, and X_c_ is the center of the Gaussian fit, respectively, while w is the value of the full width at half maximum, describing the difference between the two values of an independent variable, where the dependent variable is equal to the half of its maximum.

(3) $$y=y_{0}+\frac{Ae\frac{-4\;\ln\left(2\right){\left(x-x_{c}\right)}^{2}}{w^{2}}}{w\sqrt{\frac{\pi }{4\;\ln\left(2\right)}}}$$

### Fluorescence quenching measurements

Fluorescence quenching of the tryptophans of G-actin and Myo16Tail was measured by using acrylamide as a quencher. The steady-state fluorescence quenching was obtained using HORIBA Jobin Yvon Fluorolog 3.22 spectrofluorometer. The fluorescence lifetime quenching was measured with HORIBA Jobin Yvon Nanolog spectrofluorometer (HORIBA Scientific). The fluorescence intensities of tryptophans were determined at 350 nm for the Stern–Volmer analysis. Data were corrected with the inner filter effect during the analysis. Origin 2020 software was used for data evaluation using the Stern–Volmer model for steady-state fluorescence quenching and for lifetime quenching according to Equation 4 and Equation 5, respectively (62), where F_0_ and F are the fluorescence intensities or τ_0_ and τ are the fluorescence lifetimes of tryptophans in the absence and presence of the quencher, respectively; K_sv_ is the Stern–Volmer quenching constant and [Q] denotes the quencher concentration.

(4) $$\frac{F_{0}}{F}=1+K_{SV}\left[Q\right]$$

(5) $$\frac{\tau_{0}}{\tau }=1+K_{SV}\left[Q\right]$$

### TCSPC

Fluorescence lifetime and anisotropy of tryptophan residues were measured by the means of TCSPC using a HORIBA Jobin Yvon Nanolog spectrofluorometer. The excitation was performed by a 295-nm NanoLED as the light source (HORIBA Scientific), and the fluorescence emission was measured at 350 nm. Further experimental parameters are as follows: channel width 0.055 ns/channel, slit 10 nm for anisotropy decay and 3 nm for lifetime measurement, detector voltage 950V, measurement range 200 ns, repetition rate 1 MHz, pulse duration <1 ns, and sync delay 50 ns. Time-resolved lifetime and anisotropy decay measurements were performed with 5 to 5 μM of G-actin and Myo16Tail by adding GuHCl (1, 2, 3, 4 M). Time-resolved fluorescence lifetimes (τ) were calculated according to Equation 6, where I(t) is time-dependent intensity, IRF is instrument response function, A_i_ amplitude of the i-th component at time zero, and τ_i_ denotes for lifetime of the i-th component. Data were analyzed with exponential model (reconvolution) by FluoFit software (PicoQuant, Berlin, Germany).

(6) $$I\left(t\right)=\int_{-\infty }^{t}IRF\left(t^{\prime }\right){\sum_{i=1}^{n}A_{i}\cdot e}^{-\frac{t-t^{\prime }}{t_{i}}}dt^{\prime }$$

Time-resolved anisotropy data were fitted by Origin 2020 software using two-exponential fit, and rotational correlation (θ) values were derived from Equation 7, where r(t) is time-dependent anisotropy, r_∞_ is anisotropy at t = ∞ (limiting anisotropy), A_i_ is the pre-exponential factor of the i-th component, and θ_i_ indicates the rotational correlation time of the i-ith component.

(7) $$r\left(t\right)=r_{\infty }+\sum_{i=1}^{n}A_{i}\cdot e^{-\frac{t}{\theta_{i}}}$$

### CD spectroscopy

Far-UV (190–250 nm) CD spectrum of Myo16Tail was recorded in 50 mM Na_3_PO_4_ buffer (pH 8.0) containing 100 mM NaCl at 25 °C using a Jasco J-810 spectropolarimeter (JASCO) equipped with a Peltier-type temperature control. The applied concentration of Myo16Tail was 12.7 μM (1.1 mg/ml), which was measured by the absorbance at 280 nm. The experimental parameters were as follows: path length 0.01 cm, bandwidth 1 nm, scanning speed 20 nm/min, and response time 4 s; six scans were accumulated. The far-UV CD spectrum was corrected for the baseline by subtracting the CD spectrum of the buffer measured under the same conditions. The CD spectra were analyzed by the BeStSel (85, 86) webserver (http://bestsel.elte.hu) for secondary structure composition. Thermal denaturation experiments were carried out at a concentration of 1.15 μM (0.1 mg/ml) in 50 mM Na_3_PO_4_ (pH 8.0) containing 20 mM NaCl in a 1-mm cell. Spectra were recorded in the temperature range of 10 to 100 °C with 10 °C steps accumulating three scans and using a heating rate of 1 °C/min between temperature points of spectrum collection. The final CD spectra were plotted by using Origin 2020 software.

## Data availability

Data are available in the supporting information. All remaining data are contained in the article.

## Supporting information

This article contains supporting information (89).

## Conflict of interest

The authors declare that they have no conflicts of interest with the contents of this article.
