# Supplementary material for: The C-terminal tail extension of myosin 16 acts as a molten globule, including intrinsically disordered regions, and interacts with the N-terminal ankyrin
Source: J Biol Chem. 2021 Apr 28;297(1):100716. doi: 10.1016/j.jbc.2021.100716 (PMC8253979; doi:10.1016/j.jbc.2021.100716)
Supplement: Figures S1 to S4 and Tables S1 and S2 [file mmc1.docx]

**Supporting Information**

**The C-terminal tail extension of myosin 16 acts as a molten globule, including intrinsically disordered regions, and interacts with the N-terminal ankyrin**

Elek Telek^1,4^, Kristóf Karádi^1,2^, József Kardos^3^, András Kengyel^1,2,4^, Zsuzsanna Fekete^1^, Henriett Halász^1,4^, Miklós Nyitrai^1,2,4^, Beáta Bugyi^1,2*^ & András Lukács^1,2,4*^

^1^ Department of Biophysics, Medical School, University of Pécs, Pécs, H-7624, Hungary
^2^ Szentágothai Research Center, Pécs, H-7624, Hungary
^3^ Department of Biochemistry, Institute of Biology, Eötvös Loránd University, Budapest,

H-1117, Hungary
^4^ MTA-PTE Nuclear-Mitochondrial Interactions Research Group, Pécs, H-7624, Hungary

**This files includes:**

Figure S1. Multiple sequence alignment of Myo16Tail

Figure S2. Time-resolved lifetime decay of free Alexa568 dye and Alexa568–Myo16IQ

Figure S3. Steady-state fluorescence emission of free Alexa568 and Alexa568–Myo16IQ

Figure S4. Analysis of far-UV CD spectrum of Myo16Tail in terms of double wavelength plot

Table S1. Tryptophan fluorescence lifetime of acrylamide quenching

Table S2. Tryptophan fluorescence lifetime and rotational correlation time of TCSPC measurements

**:::**: ****:* :****:: ::: :::* :: *:* *::. ::*. ******:*****: ::**

Hs 1 LQRKIITCQKVIRGFLARQHLLQRISIRQQEVTSINSFLQNTEDMGLKTYDALVIQNASDIARENDRLR

Mm 1 LQRKIVTCQKVIRGFLARQHLLQRMSIKQQEVTSIKSFLQSTEDMALKTYDALVIQNASDIAREHDRLR

Rn 1 MQRKIVTCQKVIRGFLARQHLLQRMSIKQQEVTSIKSFLQSTEDMALKTYDALVIQNASDIAREHDRLR

Gg 1 LQKKIITCQKVVRGFLARQRVLQKMSIKQQEITSVKGFLQNVEDMGLKTYDALVIQNASDIARENDRIR

Xt 1 LQKKIILCQKVIRRFLARQHLQKKLDTKENEDTFIKDFIQTIENMGQQSYHSLVIQNASDIAREKVALQ

Dr 1 LHRKIIICQKVMRGWLARQQVRRRLTSRKQELCNVQRFLQGAEDLGLRTYDNLVIQNAADIARENDRLR

**. :. *. :: .. :.:. . : .***

Hs 70 SEMNAPYHKEKLEVRNMQEEGSKRTDDKSG-------------PRHFHPSSMSVCAAVDGLGQCLVGPS

Mm 70 KEVHTAYH------RNRQEEGTKRAEDQGG-------------CRHVHSNSVPVPMVVDSLAQALTGPS

Rn 70 KEVHAAYH------RNRQEEGTKRAEDQGG-------------CRHAHSNSVPVPMAVDSLAQALAGPS

Gg 70 NEMNAAYHREKLEARNRPEEVHKRAEDKGGKLPEDGF-AGYRAPKHFHSSSVPVPMAVEGLVHSAAGSS

Xt 70 IESNTAYS--------AMESICNKEEMLKR-------------YSLFFSSFLPLQIVSN-----IALSS

Dr 70 GHISAPPLGERPEPVGKEEDSPKRVVEKSGRGNDVANRGGNRPIRHFRSSSVPLPLVMDSLVHSSIGAS

**. : * ::.. * ************:*.************ * : : *.*::*******

Hs 125 IWSPSLHSVFSMDDSS--SLPSPRKQPPPKPKRDPNTRLSASYEAVSACLSAA-REAANEALARPRPHS

Mm 119 TRPPSLHSVFSMDDST--GLPSPRKQPPPKPKRDPNTRLSASYEAVSACLSAA-KDAAGEALTRPRPHS

Rn 119 SRSPSLHSVFSMDDST--GLPSPRKQPPPKPKRDPNTRLSASYEAVSACLSAT-KDAASEALTRPRPHS

Gg 137 IRSPSLHSVFSMEDNS--SLPSPRKQPPPKPKRDPNTRLSASYEAVSACLSAASKEAANEVLTRPRPHS

Xt 112 IRSLSLHSVLSMDDGN--CLQSPRKQPPPKPKRNPTTRLSASYEAVSACLFAASVDIDNEALSRPRPHS

Dr 139 IKAALQPTPHSTDEGNGGSLSSPRKQPPPKPKRDPNTRLSASYEAVSAGLSIAPKDSPAEALAKPRPHS

************* ******:***:.*:*** .. :.: .**

Hs 190 DDYSTMKKIPPRKPKRSPNTKLSGSYEEISGS-RPGDARPAG----APG------AAARVLTPGTPQCA

Mm 184 DDYSTMKKIPPRKPKRSPHTKLSGSYEEIWGPPRPSGTMGQGGRHQAPGTLSVQWARPDSVPQCTPQLP

Rn 184 DDYSTMKKIPPRKPKRSPHTKLSGSYEEIWGP-RPSGTMGQVGKHHAPGTLGVQWASPDSMPQCTPQLP

Gg 203 DDYSTMKKIPPRKPKRSPNTKLSGSYEEIPGQ-KPGDVKQANTVAKQGS-----YDTAGVQRAASADVP

Xt 178 DDYSTMKKIPPPKPKRSPNTKLSSSFEEISAH-NSGELNTFSTIVRKVHR-----DIGTIQRAASADGP

Dr 207 DDYSTMKKIPPPKPKRSPNTKLTGSYEEISVP-SPARPTDMKLVSLVRGG----HCLGLIQRAASADGP

**. : : * ***** * : **: **:****** :.**

Hs 247 LP--PAAPPGD-EDDSEPVYIEMLGHAARP-----DSPDPGESVYEEMKCCLPDDGG-----PGAGSFL
Mm 252 LH--LPLPQGDYDDDAEPVYIEMVGNAARAGGSETDSPDQGESVYEEMKYILPEEG------CGLGMLT
Rn 251 LH--LPLPQGDYDDDGEPVYIEMVGNAARAGGSETDSPDQGESVYEEMKYVLPEEG------CGPGMLT
Gg 265 QHGTLSLYMSQEEEENEPVYIEMVGNAMKHSSAEAESPEQGESVYEEMKYFLPEEGI-----NSNGIIP
Xt 240 HHGILTLCMSEEEDEAEPVYIEMAGTAGKCHFIERSSPEQGEAVYEEMKYHHPDHI---------NINN
Dr 270 HSSVLSLYPCQ---DEEDVYIEMVGASRALSLADTHSPEPGEAVYEEMKYFPNDEIVSTPVVKPEAVNP

**:. * : .****.*****************:.** ******

Hs 303 LHGASPPLLHR-------APEDE-----AAGPPGD--ACDIPPPFPNLLPHRPPLLVFPPTPVTCSPAS

Mm 312 FLPASPPLFLETRKAIILEAAEG-----NCQPSKD--TCDIPPPFPNLLPHRPPLLVFPPTPVTRSPAS
Rn 311 FLPASPPLFLETRKAIILEAGEG-----SCQPLKD--TCDIPPPFPNLLPHRPPLLVFPPTPVTCSPAS

Gg 328 VATGSPPLVFESKKNVHVEDGTLDGNSQAALNYKD--SCDIPAPFPNLLPHRPPLLVFPPTPVTCSPAS

Xt 299 LAMGSSLLQNMNLATFENLDADIK----NEQCSKE--SCDIPAPFPNLLPHRPPLLVFPPATVTSSPAS
Dr 335 TPLPAPVLEIKRPVTLIEQSGTLG----GKQQGKDGMPCDIPAPFPNLLPHRPPLLVFPPSPVTCSPAS

***********: *****:**::*. * : .**:*.* : : :**.: :: .: :** *****

Hs 358 DESPLTPLEVKKLPVLETNLKYPVQPEGSSPLSPQYSKSQKGDGDRPASPGLALFNGSGRASPPSTPPP

Mm 373 DESPLTPLEVKKLPVLETNLKYPVQSEGSSPLSPQYSKAQKGDNDQLASPGFPVFNGPSRISPPATPPP

Rn 372 DESPLTPLEVKKLPVLETNLKYPVQSEGSSPLSPQYSKAQKGENDQLTSPGFPVFNGPSRISPPATPPP

Gg 394 DESPLTPLEVKKLPVLETNLKYPVQSEGSSPLSPQYSKSQKGESERPASPGLVVFNVSSKVTPPSTPPP

Xt 361 DESPLTPLEMIKLPVLETNINYTIQTDMSSPISSQCLKHQR-DNERPSSPALSVFSVSSKVSPPCTPPL

Dr 399 DESPLTPLEVKKLPVFETNLNYSSQ-DGGSPLSPQYTRQRA-----DSSPSLSILMP-DKSTPPLTPPP

*** *. . : :*:.** :**

Hs 358 PPPPP----GPP------------------------------------------PAPYRPCAHLAFPPE

Mm 441 PPGPPPAPCGPPPAPCGPPPAPCGPPPAPCGPPPAPCGPPPAPCGAASASCGVAPAPCRPPTHFAFPPE

Rn 440 PPGPPPAPCGPPSAPCGPPPAPCGPPPVPCGPPPAPCGPPPAPCGAA-------PAPCRPPTHFAFPPD

Gg 462 PLPPPPPPVPPP-------------------------------------------ISYRASTHFAFPPE

Xt 428 PQTPT---------------------------------------------------SFQFPTHFSFPME

Dr 460 PPPPAVLP-----------------------------------------------PPYRPPSHFPFPPE

**: ..:: : : . . . * .:*. :. *.. . ..**

Hs 372 ---PAPVNAGKAGPSAEAPKVHPKPNSAPVAGPCSSFPKIPYSPVKATRADARKAGSSASPPAPYSPPS
Mm 509 ---SVLVTAAKALTNSDLPRTQPKPSSAPVPGPCSSFVKAPYSPGKTARADLRKTSSTFSPPSPYSPPN

Rn 501 ---SVLVTAAKALTNSDLPRTQPKPSSAPVLGPCSPFVKAPYSPGRTARADLRKASSTFSPPSPYSPPN

Gg 487 PCASFLINTGKTGTNSDLSKVPQRPNPAQLGCSPSPFSKLPYSP-KVARAEHKKLNSNSSSSFPYSPTN

Xt 445 SSYALLVGKNLKTTNADSVKIAPKSNAVITEESCGSLTKLTFSSPIKNARTMQNKSHLGSPSLATSLTN
Dr 481 PN-FLALTRAASVASTESPKVSHKMGHGLAETP-PSSSKPPFSPVKTSRPEPRRAHSCSSSPLLFNPAN

**::****** ::*.****:**:*:.**:**: : *:*: . :: : **.****:**

Hs 437 SRPLSSPLDELASLFNSGRSVLRKSAAGRKIREAEGFETNMNISSRDDPSTSEITSETQDRNANNHGIQ
Mm 574 SRPLSSPLDELASLFNSGRSVLRRSAVGRRIREAEGFETNMNLSSRDEPSSSEMASETQDRNANNHGTQ

Rn 566 SRPLSSPLDELASLFNSGRSVLRRSAVGRRIREAEGFETNMNLSSRDEPSSSEMASETQDRNANNHGTQ

Gg 554 SRSLTSPLDELASLFNSGRSVLRKSAAGRKIREPEGGETNLNLRSREDPNASDTGSETQDKNANNHGTQ

Xt 513 ISHITSPLDELTTLFSSGRSLLRKSAAGRKIREQEGVEMNLNVCGGEDVAMP----DTQDNNANNQDNR

Dr 547 GRPLTSPLDELNTIFSSGRSLLRKSTTGRKIRDSGLSNSNINLPGREDQGPISPSAQLQDKNANNHTSP

**. *. . **** ::** . ..::.: ... ***

Hs 505 LSNSLSSAITAENGNSISNGLPEEDGYSRLSISGTGTSTFQRHRDSHTTQVIHQLRLSENESVALQELL

Mm 642 LSSSLSSDVTAENGNPVTNGLAEDDGCSRLCLSGMGTSSFQRNRESHTTQVIHQLRLSENESVALQELL

Rn 634 LSSSLSSVVAAENGNPVTNGLAEDDGCSRLCLSGMGTSSFQRHRESHTTQVIHQLRLSENESVALQELL

Gg 622 SSNPLSSSVTAENGNSVSNGVPEENEYSRLSASTTAGSSIQRHRESHTTQVIHQLRLSENESVALQELL

Xt 577 GPNSRSSCAAFENGNSVTNG-------EYFTLENYCTKKIRNKRKNFFFQVIHQLRLSKNESAALQELM

Dr 615 NS---SNPAPIENGNQISNGTLEDEGHPKPMAS--SSSTLHRHMDSHHTQ-------------------

Hs 573 DWRRKLCEEGQDWQQILHHAEP--RVPPPPPCKKPSLLKKPEGASCNRLPSELWDTTI

Mm 710 DWRRKLCEAREGWQEALQHPEP--RAPPPPPCKKPTLLKKPEGGSCTRLPSQLWDSSM

Rn 702 DWRRKLCESREGWQEAMQHPEP--RAPPPPPCKKPTLLKKPEGGSCTRLSSQLWDSSI

Gg 690 DWRRKLCEEREDWQQILHNTEQRITAPPPPPCKKPTLLKKVEDASCNRLSSGIWDTTM

Xt 638 DWRRKLCEGNGEWQTEQRNSAS----------THSS----------------------

Dr 659 ----------------------------------------------------------

**Figure S1.** Sequence comparison of Myo16Tail from different vertebrate species. Multiple sequence alignment was carried out by Clustal X. Accession numbers: *Homo sapiens (Hs*): Q9Y6X6, *Mus musculus (Mm)*: Q5DU14, *Rattus norvegicus (Rn)*: Q9ERC1, *Gallus gallus (Gg)*: XP_416950.3, *Xenopus tropicalis (Xt*): A0A5G3IJG7, *Danio rerio (Dr)*: F1QE80. Sequences and amino acid conservations are indicated and shaded as follows: fully conserved – black (*), strongly similar – dark grey (:) and weekly similar – light grey (.). The red arrows show the positions of phosphorylation sites predicted by PhosphositePlus. The known sequence motifs are indicated in boxes: IQ (grey), NHM (orange) and proline-rich (green).

**

**

**Figure S2.** Time-resolved lifetime decays of free Alexa568 dye and Alexa568-labeled Myo16IQ using time-correlated single photon counting. The excitation wavelength was 570 nm (fixed excitation wavelength of the LED) and the emission was detected at 601 nm using slits 5-5 nm. The lifetime data (mean ± SD, n = 3) was calculated according to Equation 6. Derived average lifetime of free Alexa568 was 2.84 ± 0.006 ns and for Alexa568–Myo16IQ was 3.66 ± 0.030 ns. The raw lifetime decays are visualized in inset. The increasing lifetime decay confirms the labeling reaction.

**

**

**Figure S3.** Steady-state fluorescence emission of free Alexa568 dye and Alexa568–Myo16IQ. Alexa568 was excited at 578 nm and the emission was recorded between 540-700 nm using 5-5 nm slits. The maximum wavelength plot revealed a slight spectral shift from 596.3 ± 0.57 nm to 599 ± 1.00 nm (mean ± SD, n = 3) confirming the labeling reaction of Myo16IQ. The fluorescence emission spectra are visualized in inset showing the moderate shift in the emission maxima with vertical dotted lines between the free Alexa568 and Alexa568–Myo16IQ spectra.

**
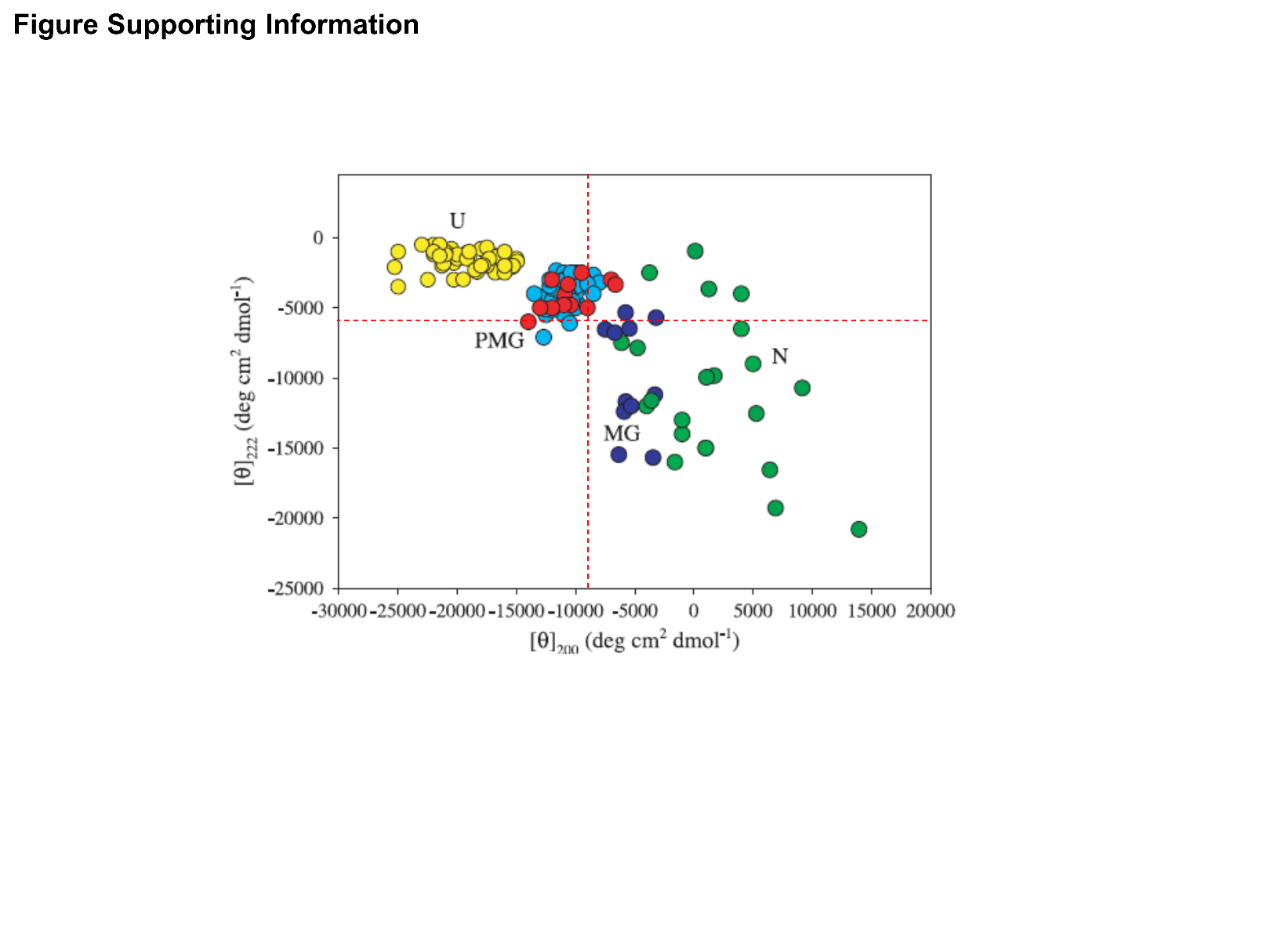
**

**Figure S4.** Analysis of far-UV CD spectrum of Myo16Tail in terms of mean residue ellipticity as a double wavelength plot, [θ]_222_ against [θ]_200_, modified based on Uversky & Fink 2004 (89). The different subclasses of conformational states of proteins are shown on the plot, native (green-N), molten globule (dark blue-MG), pre-molten globule (cyan-PMG), amyloidogenic intermediates (red in the subclass of PMG) and random coil-like unfolded proteins (yellow-U). The derived mean residue ellipticity of Myo16Tail were [θ]_222_= -5813 and [θ]_200_= -8865 deg cm^2^dmol^-1^ followed by marking the values on the plot as dashed red lines. The intersection point classifies Myo16Tail at the edge of PMG, or rather between PMG and MG subclasses of protein conformation verifying a molten globule-like conformation.

**Table S1.** Tryptophan fluorescence lifetime (τ) of acrylamide quenching of G-actin (control) and Myo16Tail derived from TCSPC measurements. Mean ± SD (n = 3).

| **Acrylamide** | **G-actin** | |  | **Myo16Tail** | | |  |
| --- | --- | --- | --- | --- | --- | --- | --- |
| **c (M)** | | **τ (ns)** | **± SD** | |  | **τ (ns)** | **± SD** |
| **0** | | 3.6 | 0.00 | |  | 4.1 | 0.00 |
| **0.26** | | 2.2 | 0.08 | |  | 3.2 | 0.08 |
| **0.48** | | 1.7 | 0.12 | |  | 3.0 | 0.12 |
| **0.67** | | 1.4 | 0.00 | |  | 2.8 | 0.00 |
| **0.83** | | 1.3 | 0.01 | |  | 2.8 | 0.01 |
| **0.97** | | 1.2 | 0.01 | |  | 2.8 | 0.01 |
| **1.09** | | 1.1 | 0.01 | |  | 2.9 | 0.01 |

**Table 2.** Tryptophan fluorescence lifetime (τ) and rotational correlation time (θ) values of G-actin (control) and Myo16Tail in the absence and presence of GuHCl derived from TCSPC measurements. Mean ± SD (n = 3).

| **GuHCl** | |  | **G-actin** | |  |  | |  | **Myo16Tail** | | | |  |
| --- | --- | --- | --- | --- | --- | --- | --- | --- | --- | --- | --- | --- | --- |
| **c (M)** | **τ (ns)** | **± SD** | **θ (ns)** | **± SD** | | **τ (ns)** | **± SD** | | **θ_1_ (ns)** | **± SD** | **θ_2_ (ns)** | **± SD** |  |
| **0** | 4.3 | 0.16 | 25.9 | 1 | | 4.3 | 0.08 | | 1.2 | 0.2 | 33 | 1 |  |
| **1** | 4.5 | 0.23 | 32.7 | 1 | | 4.1 | 0.19 | | 1 | 0.4 | 31 | 3.5 |  |
| **2** | 4.7 | 0.31 | 30.4 | 2 | | 3.7 | 0.15 | | 0.9 | 0.4 | 41.7 | 10 |  |
| **3** | 3.6 | 0.23 | 19.4 | 1 | | 3.4 | 0.18 | | 0.7 | 0.4 | 25.1 | 4 |  |
| **4** | 3.4 | 0.23 | 15.8 | 1 | | 3.2 | 0.12 | | 1 | 0.3 | 22.4 | 3.6 |  |
